# VariantscanR: an R-package as a clinical tool for variant filtering of known phenotype-associated variants in domestic animals

**DOI:** 10.1186/s12859-023-05426-6

**Published:** 2023-08-01

**Authors:** Fréderique Boeykens, Sofie F. M. Bhatti, Luc Peelman, Bart J. G. Broeckx

**Affiliations:** 1 Laboratory of Animal Genetics, Department of Veterinary and Biosciences, Faculty of Veterinary Medicine, Ghent University, Heidestraat 19, 9820 Merelbeke, Belgium; 2 Small Animal Department, Faculty of Veterinary Medicine, Ghent University, Salisburylaan 133, 9820 Merelbeke, Belgium

**Keywords:** Sequencing, Variant filtering, Precision medicine, Clinical tool, WES, WGS

## Abstract

**Background:**

Since the introduction of next-generation sequencing (NGS) techniques, whole-exome sequencing (WES) and whole-genome sequencing (WGS) have not only revolutionized research, but also diagnostics. The gradual switch from single gene testing to WES and WGS required a different set of skills, given the amount and type of data generated, while the demand for standardization remained. However, most of the tools currently available are solely applicable for human analysis because they require access to specific databases and/or simply do not support other species. Additionally, a complicating factor in clinical genetics in animals is that genetic diversity is often dangerously low due to the breeding history. Combined, there is a clear need for an easy-to-use, flexible tool that allows standardized data processing and preferably, monitoring of genetic diversity as well. To fill these gaps, we developed the R-package variantscanR that allows an easy and straightforward identification and prioritization of known phenotype-associated variants identified in dogs and other domestic animals.

**Results:**

The R-package variantscanR enables the filtering of variant call format (VCF) files for the presence of known phenotype-associated variants and allows for the estimation of genetic diversity using multi-sample VCF files. Next to this, additional functions are available for the quality control and processing of user-defined input files to make the workflow as easy and straightforward as possible. This user-friendly approach enables the standardisation of complex data analysis in clinical settings.

**Conclusion:**

We developed an R-package for the identification of known phenotype-associated variants and calculation of genetic diversity.

**Supplementary Information:**

The online version contains supplementary material available at 10.1186/s12859-023-05426-6.

## Background

While single-gene testing is suitable for diseases with clear symptoms and without locus heterogeneity, the results are often less or even non-informative if clinical features are not pathognomonic [1]. This problem becomes even more cumbersome for diseases with locus heterogeneity as well as complex hereditary diseases. As a consequence, patients are often subjected to multiple clinical workups, imaging and biochemical laboratory tests, which is termed “the diagnostic odyssey” [2–4]. Whole genome sequencing (WGS) and whole exome sequencing (WES) have shown to potentially provide solutions in these challenging cases. Sequencing hypothesis-free, meaning here not being focused on e.g. specific candidate genes, they are particularly useful for the diagnosis of rare or complex hereditary disorders [4, 5]. Based on the proportion of the genome sequenced, the number of variants identified with WGS is obviously higher than with WES, the diagnostic rates appear to be comparable however [6] and exceed the more traditional methods by far. A study in 2017, performing clinical WES on 1000 individuals showed for example an increase by 30.7% in diagnostic yield [7]. These properties have resulted in the extensive use of this technique in human medicine for diagnosis [3, 8–11].

WES designs are also available for other species, such as rats, cats and dogs [12–14]. As WES has already demonstrated to be a valuable tool in veterinary medicine for the discovery of putative disease-causing variants [13–15], the gradual introduction of WES in routine diagnostics for domestic animals for the same reasons as in human genetics is likely. The introduction of WES and WGS in a routine clinical diagnostic setting is however hampered for several reasons. Originally, this was related to the considerable costs, the more limited resources of accurate reference genomes compared to humans and specific set of skills necessary to properly handle sequence variant data [16]. As costs are decreasing rapidly and high-quality reference genomes for a wide range of domestic animals are now available, it is mainly the bioinformatics problem that remains [17]. In more detail, 17 out of 22 of the laboratories of the U.S. Department of National Animal Health Laboratory Network recently reported in a survey that their bioinformatic knowledge was either beginner or intermediate [17]. Any software developed should thus be easy-to-use and optimally require only basic programming skills. Practically, we translated this here to ensure that all the functions necessary for handling the data are provided and that the end user only has to run them in the correct order. While one option might be to try to implement the software tools available for human analysis, for most of the tools, this will not be possible. Furthermore, even in human medicine, a lot of the workflows actually contain custom-made scripts [18], which is of course less ideal in terms of standardization and still requires specific programming skills. It is thus clear that there is a need for a universal non-species-specific tool that ensures a standardized workflow. A final problem that is of paramount importance in veterinary medicine, is the limited genetic diversity observed in various species. This is very pronounced in the dog, a species with an incredible phenotypical diversity, but also an astonishingly low genetic diversity. This decrease in diversity is a direct result of strong selective breeding practices, such as inbreeding and the use of a limited number of popular sires [19]. As similar observations are reported in various other species, an omnipresent characteristic when working in the field of animal genetics is that genetic diversity should be monitored and, whenever possible, increased as a further reduction would only increase the frequency of disease-associated variants, the proportion of homozygotes in a population and thus disease prevalence, negatively impacting animal welfare [20]. This highlights the need for breeding programs tailored to improve genetic diversity and preventing heritable diseases. WGS or WES data can help to achieve this.

With this in mind, we developed variantscanR, an R-package written for the easy and straightforward identification of known phenotype-associated variants from the large collection of variants present in dogs and other domestic animals. This R-package aims to meet the requirements (Table 1) to be used as a tool in (veterinary) clinical genetics, in general and for genetic counselling, more specifically.

**Table 1:** The software tool criteria that should be met for solving the practical questions regarding diagnostics, genetic diversity and breeding policy discussed in this article. The tool was developed to enable a widespread use of WES/WGS data in veterinary clinical genetics

Criterium 1	Extract all variants that are phenotypically and/or clinically relevant from sequencing data
Criterium 2	Work with data formats that are standardly used
Criterium 3	Create a standardized report
Criterium 4	Analyze non-human data
Additional criterium 1	Report genetic diversity

## Implementation

To qualify as an easy-to-use, straightforward, bioinformatic tool in veterinary medicine, variantscanR must provide flexibility to serve the *modus operandi* of each laboratory or clinic.

An overview of the features of the clinical tool is given below. Furthermore, extra information is provided in the package vignette, which can be found on the package website and in the supplementary material (Additional file 1). An example of each discussed input file in its original format is made available on https://github.com/FrederiqueBoeykens/variantscanR_extra.

### Workflow

#### Keeping it simple: the two-step-three-files approach

To keep the workflow as straightforward as possible, one can go from input to results with only two functions and three input files. These functions encompass the entire process, from opening the input file into the R environment and the processing of data, to the creation of the output file. However, it is a well-known fact that many different formats are used in bioinformatics. The same kind of data are thus often represented in various ways. Just one example are chromosome notations. The package provides several auxiliary functions for these purposes, because it is assumed that a majority of the target users will not have an in-depth bioinformatics background which is often required to understand and especially to manipulate these formats. First, the two-step-three-files-approach will be explained, after which the additional steps will be elaborated on.

**Fig. 1:**
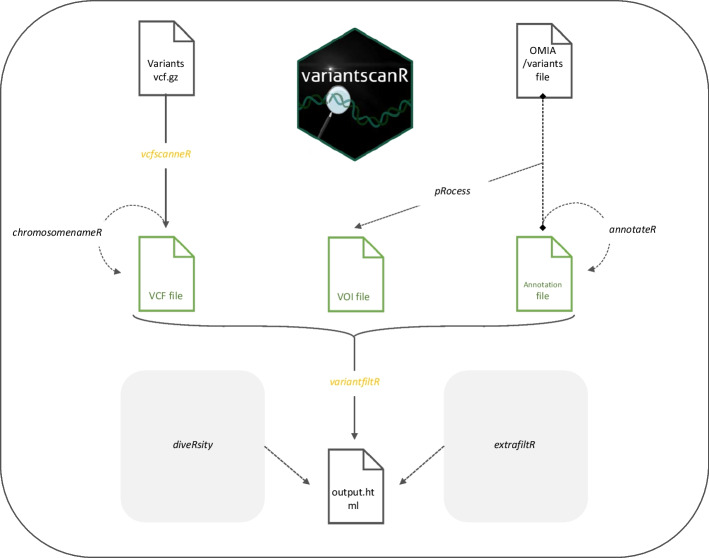
Overview of the variantscanR workflow. Files are shown as file icons, functions are written in italic. The functions required for the two-step workflow are depicted in yellow. The three input files needed for the two-step workflow are depicted in green. These are the variant call format (VCF) file, the variants of interest (VOI) file and an annotation BED file. The grey functions are additional, optional functions that make the package as user-friendly as possible. Next to these additional functions, two supplementary functions were created that are out of the variant-filtering scope of the workflow: one allows the calculation of the genetic diversity of every individual sample in a multi-sample VCF file, while the second function retains all the remaining variants present in the sample for the genes of interest

An overview of the workflow is provided in Fig. 1.

##### Input files

Overall, the pipeline supported by variantscanR starts from the standard variant call format (VCF) file. Because the VCF file format is not one of the standard formats that can be uploaded in the R environment, a specific function was created. The *vcfscanneR* function not only uploads a VCF file but also converts the file into a suitable format for downstream analyses, which makes this a mandatory step before filtering (Fig. 1).

While obtaining a VCF file is either way a prerequisite to process variants, two other files are needed for the second part of the two-step approach. The first file is a data file containing the phenotype-associated variants of interests (VOI) in a specific format, i.e. the chromosome, the location, and the gene in which the variant is located have to be included. A detailed description is available in the package vignette (Additional file 1). The second file is an annotation file that contains information about the gene, its transcripts, the number and length of the exons. This information is used for annotating the user-defined file with the variants of interest and for appropriate reporting in clinical settings.

##### Filtering

The filtering itself is achieved by the *variantfiltR* function. This function takes the single sample VCF file, obtained after the *vcfscanneR* function, and filters the variants of interest present in the sample. Because this step is the most time-consuming, a process bar is displayed. After filtering.html files are obtained.

##### Output

The output files are raw.html files that are saved in the current working directory. These files are also opened in a tab of the default web browser and will pop up automatically. In total, 5 tables, and thus 5 raw.html files, will be generated and are combined in an interactive report. Two of the 5 tables offer concise breeding advice (Tables 2, 3). The other three tables list the variants that were found in the sample. The most important table (Table 4) contains the variants that were found in a hetero- or homozygous state in the sample and that are known to segregate in the breed of interest. These variants have an established genotype–phenotype association in that breed, making them the most important variants to report. The variants that were identified in the sample, but that were not known to segregate in that breed, are reported in a separate table (Table 5). As these were not known to segregate, their presence requires specific attention and extra careful reporting: it might be that the genotype–phenotype association is the same, but was not yet established, but the association with the phenotype might also be breed-specific, which has already been reported [21–23]. The last table (Additional file 2) consists of the variants found in homozygous wild type state, meaning these variants were called in another sample of the multi-sample VCF file. While thus less important for the phenotypes or clinical decision making for that specific individual, this table is important 1/ to know which variants segregate in other individuals in your population and 2/ when checking which animals are suitable mating partners.

**Table 2:** Breeding advice for phenotype-associated non-reference variants that occur in a homozygous state

Inheritance pattern	Breeding advice
Autosomal dominant	Animal can NOT be used for breeding purposes. Offspring would be carrier or homozygous and might develop symptoms
Autosomal recessive	ONLY combine with wild type animal! Offspring will be carrier and, on its turn, can ONLY be combined with wild type animal ***
Mitochondrial	If animal is female: animal can NOT be used for breeding purposes. Mitochondria are inherited maternally
X linked dominant	Do NOT use animal for breeding purposes because all offspring will inherit the defective X chromosome
X linked recessive	Do NOT use animal for breeding purposes as all male offspring will inherit the defective X chromosome and will thus be affected
NA	Not able to provide breeding advice because inheritance pattern was not included in the input file

***Important general side note: Do NOT use animal if this would harm the welfare of the animal itself, or the progeny. Furthermore, the animal must be capable of carrying out a healthy pregnancy

**Table 3:** Breeding advice for phenotype-associated non-reference variants that are found to be heterozygous

Inheritance pattern	Breeding advice
Autosomal dominant	Animal can NOT be used for breeding purposes
Autosomal recessive	Animal can be used for breeding purposes but ONLY if combined with a wild type animal. Offspring will be ± 50% carrier and ± 50% wild type
Mitochondrial*	If animal is female: animal can NOT be used for breeding purposes. Mitochondria are inherited maternally. If animal is male: animal can be used
X linked dominant	If animal is female: do NOT use animal for breeding purposes because ± 50% of the offspring will inherit the defective X chromosome
	If animal is male, only one X chromosome is present. Accordingly, affected animals can NOT be used for breeding purposes as all female offspring will be carrier and thus will be affected
X linked recessive	If animal is female: animal can NOT be used for breeding purposes as ± 50% of male offspring will inherit the defective X chromosome and these male animals will develop symptoms
	If animal is male, only one X chromosome will be present. Affected animals can be used ONLY if combined with a wild type female animal. Female offspring will be carrier of the variant
Y linked	The animal is male and can NOT be used for breeding purposes as every male offspring will inherit the defective Y chromosome
NA	Not able to provide breeding advice because inheritance pattern was not included in the input file

*Mitochondrial heteroplasmy is the situation in which more than one type of mtDNA is present within a cell. Levels of heteroplasmy can vary between cells and tissues of one individual and influence the threshold for disease phenotype

**Table 4:** Priority list of variants that were found in the sample of interest and that are known to segregate in the breed (Labrador retriever) with an established genotype–phenotype association

Chromosome	Location	Gene	Reference	Allele 1	Allele 2	Zygosity	Inheritance	Phenotype
12	2,652,874	COL11A2	C	C	G	Heterozygous	Autosomal recessive	Skeletal dysplasia 2
5	63,694,334	MC1R	G	A	A	Homozygous	Autosomal recessive	Red/yellow coat
X	60,279,238	ATP7A	C	C	T	Heterozygous	X linked recessive	Menkes disease

**Table 5:** Variants in the sample of interest, but not known to segregate in the breed (Labrador retriever). This warrants additional careful interpretation as the genotype–phenotype association can be breed-specific

Chromosome	Location	Gene	Reference	Allele 1	Allele 2	Zygosity	Inheritance	Phenotype
32	4,509,367	FGF5	G	A call could not be made for this sample at this given locus	A call could not be made for this sample at this given locus	Zygosity could not be determined	Autosomal recessive	Long hair
5	63,694,460	MC1R	C	T	T	Homozygous	Autosomal dominant	Black melanistic mask

A final and important note: if no call was made for one of the loci of interest, this will be communicated in the previously mentioned tables. We consider it important that this information is not withheld from the user because the genotype for these loci is thus unknown and consequently no conclusion can be drawn as to whether the variant is present. If such information is withheld, the user may erroneously conclude that the animal does not carry this variant.

#### Making it simple: additional functions

##### Auxiliary functions: data pre-processing and quality control

As stated in the previous section explaining the two-step-three-files approach, both a data file containing the phenotype-associated VOI and an annotation file are prerequisites. While these files can be created manually, there are several auxiliary functions that automatize this process and can identify errors. The VOI file will be discussed first. This file can be created manually with details described in the vignette, however, we also wanted to ensure compatibility with existing resources: a species-specific file containing every known phenotype-associated (likely causal) variant can also be downloaded from the Online Mendelian Inheritance in Animals (OMIA) website [24, 25]. If OMIA is listed as the source of the data, the function automatically adapts the file to the desired format, eliminating the need to manually do this.

Irrespective of the source of the VOI file, user-defined files or manually edited files are prone to human errors, as such, quality control can be useful. For this reason, the *pRocess* function provides several optional quality control steps. This includes screening the given genomic locations for mismatched base pairs with a reference genome. If a mismatch is encountered, the user is notified and will be asked to manually check the location for this variant. The user can provide a reference genome version so that variants mapped to another version are not considered during filtering. Finally, rows that do not contain information about the chromosome, location and reference genome are ignored automatically.

There is also an auxiliary function to create the annotation file. Based on data downloaded from the UCSC table browser website, the *annotateR* function generates an annotation file without the need to make manual adjustments [26]. This is also explained in detail in the package vignette.

Lastly, depending on the reference genomes used for creating the VCF file, the three files might be inconsistent in terms of the chromosome nomenclature used. The *chromosomenameR* function enables the conversion, when needed. An example and useable file containing different chromosome names for dogs, is available on the package website as supplementary data.

##### Diversity

It is a well-known problem that the genetic diversity can be dangerously low in domestic animals, especially in dogs [19, 27]. That is why variantscanR also features a diversity analysis function, in addition to its main functions. The measure used to quantify diversity is the average heterozygosity, which is calculated as follows:

$${H}_{e}= \frac{{n}_{He}}{{n}_{n}}$$

With ${H}_{e}$ being the level of heterozygosity; ${n}_{He}$, the number of heterozygous loci in the sample of interest and ${n}_{n}$, the total number of loci used. To avoid estimates that differ based on the number of loci included, ${n}_{n}$ is the same for every sample of the multi-sample VCF file by only including those loci that are called across all samples. While this implies that the number of loci will decrease with more samples being included in the multi-sample VCF file, the number of loci that remain should still be substantial.

The average heterozygosity is an accessible and uncomplicated measure for within-individual heterozygosity and is easy to calculate if genotype data, such as SNPs or genetic markers are available [28, 29]. This is also an intuitive measure as a reduction in genetic diversity results in an increase of the number of homozygous loci in an individual (and as such, the probability that recessive traits will appear also increases, while the individual fitness decreases). Breeding for heterozygosity will prevent the latter by maintaining or even increasing the genetic pool [29].

To estimate the diversity of each sample as accurate as possible, only the variants that are called appropriately are withheld in the multi-sample VCF file. If there are insufficient data to determine the genotype for a particular locus in one of the individuals, this locus is not retained for the diversity calculations. If the *diveRsity* function is used, the obtained tables and graphs will be added automatically to the.*html* page. In addition, when using the *diveRsity* function, a separate folder per sample will be generated in the current working directory, containing all the sample-specific tables and graphs.

##### Extra functions: dealing with genetic heterogeneity

When there is genetic heterogeneity, the variant responsible for a certain phenotype might not be known and as such are not present in the VOI file. The disease-associated variant might however occur in genes known to be associated with the phenotype. The optional *extrafiltR* function collects all other variants present in the genes that contain the variants of interest as this might be valuable input in these cases. Again, raw.html files are created as an output. These files can be searched by the user to identify potentially interesting variants.

## Results and discussion

Because this R-package was specifically designed to allow non-human data processing, a canine example is provided.

### Example: Labrador retriever

#### Preparatory steps

WES data were obtained from 4 different Labrador retrievers for an independent project at the laboratory. The processing of one randomly selected sample is demonstrated in detail, while the output of the other three samples can be found in the supplementary data (Additional file 3). The selected sample is a healthy, yellow, female Labrador retriever.

The multi-sample VCF file was uploaded into the R environment after which some basic information, concerning the VCF itself, was rendered to the console. The latter includes information on the file attributes, such as the number of meta lines, the number of lines in the header, the number of variants and columns (Additional file 4). While the next step is not necessarily required, the *chromosomenameR* function was used for demonstrative purposes. In this specific case, the chromosome names in the VCF file needed to be altered to the ‘chrN’ notation, with N being the chromosome number for downstream analysis, to allow a match with the OMIA-downloaded VOI file.

Next, the necessary BED files were downloaded from the UCSC table browser website for annotation in the downstream analysis. The appropriate clade, genome and assembly were chosen. More information on how to obtain the necessary files and formats are available in the package vignettes (Additional file 1).

The VOI file used here contained 172 variants. Quality control with the *pRocess* function was performed on the VOI file and 4 wrongly annotated variants were manually curated. This included, for example, the adjustment from chr19 to chr29 for a variant located in the *CLN5* gene, clearly indicating the importance of quality control and that human errors are often inevitable.

#### The actual analysis

After these preparatory steps, the *variantfiltR* function was used. This function generated two groups of tables: breeding advice tables (Tables 2, 3) and tables containing variant data (Tables 4, 5 and Additional file 2).

Because the most important output table (Table 4) contains the variants for which the sample is not homozygous wild type, this table will be discussed in more detail. Variants were found in three different genes, each with multiple transcripts. The first variant is located on chromosome 12 in the *COL11A2* gene and is associated with Skeletal dysplasia 2 (SD2). SD2 is characterized by short legs while maintaining normal body length and width. Since SD2 follows an autosomal recessive inheritance pattern and the sample is heterozygous for this variant*,* there will be no observable phenotype. However, knowing the carrier status is important to give correct breeding advice for a wide range of phenotypes/diseases. Therefore, this variant is significant [30]. The second variant is located on chromosome 5 in the *MC1R* gene, which determines coat color in Labrador retrievers. A recessive mutation in this gene results in a non-functional receptor, leading to a yellow coat color. The dog in the study is homozygous for this variant and indeed has a yellow coat [31]. The final variant is located on the X chromosome. The sample is a female Labrador retriever and is heterozygous for this locus. In dogs, the mutation in the *ATP7A* gene is associated with decreased hepatic copper levels, providing protection against copper toxicosis in dogs homozygous or heterozygous for the mutation in the *ATP7B* gene. The presence of the ATP7B mutation is necessary for copper deficiency and the manifestation of related phenotypes. Without the ATP7B mutation, despite the presence of the variant in the *ATP7A* gene, no copper deficiency phenotype will be observed [32, 33].

Table 5 includes the variants that are not known to segregate in the breed but are found in hetero- or homozygous state in the sample. Lastly, Additional file 2 consists of the variants found in homozygous wild type state, meaning the variant is called in another sample of the multi-sample VCF file.

#### Diversity

**Fig. 2:**
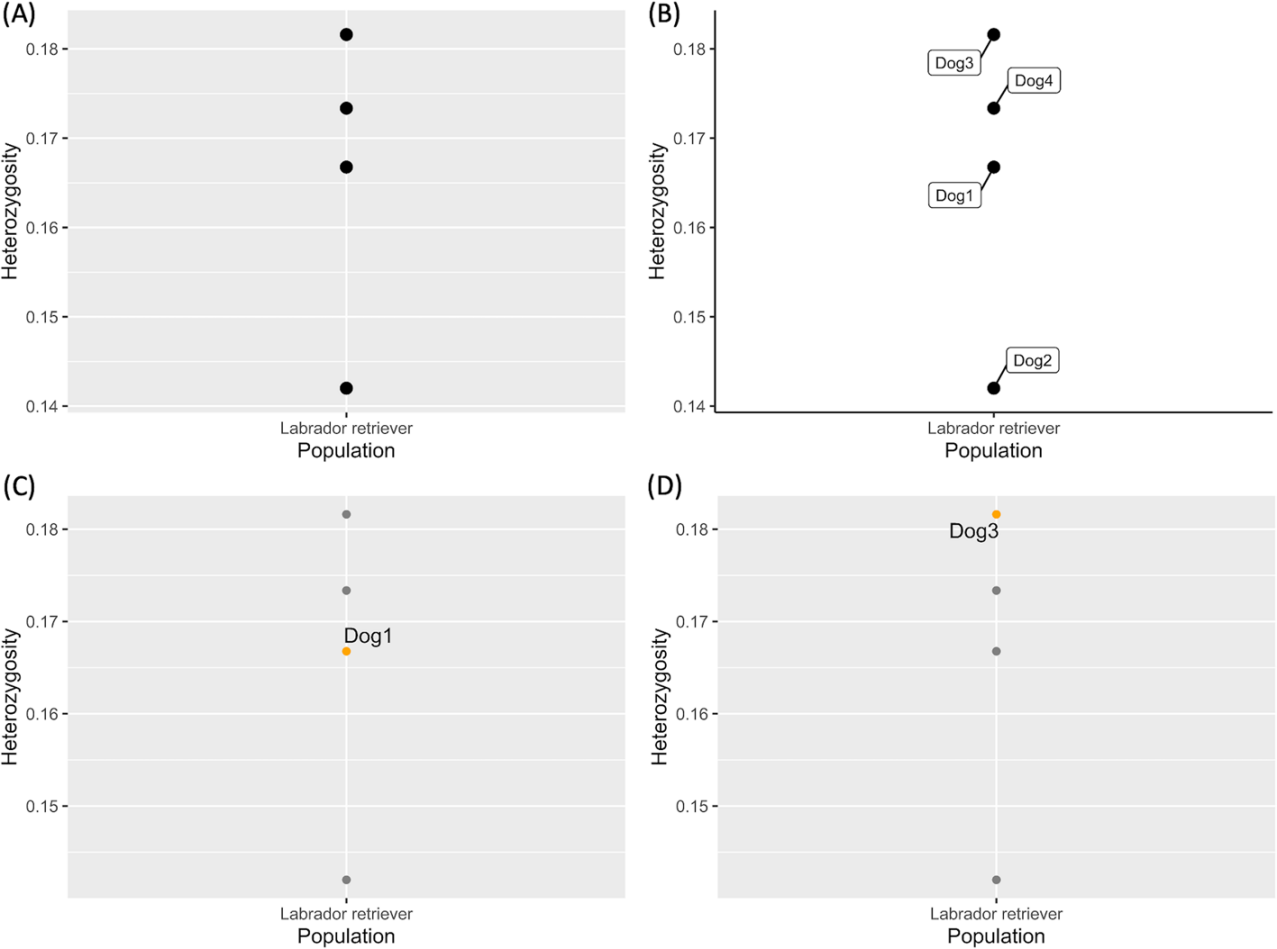
diveRsity function output. Heterozygosity per population graphs with (**A**) basic overview graph without any sample names; (**B**) fully annotated graph; (**C**) graph in which only the sample of interest is highlighted; and (**D**) graph highlighting the sample with the highest level of heterozygosity. The heterozygosity per sample H_e_ is obtained by dividing the number of heterozygous loci, n_He_, by the total number of loci used, n_n_

After analysis with the *diveRsity* function of the multi-sample VCF file, multiple graphs are generated displaying the diversity, expressed as the level of heterozygosity, of the studied animal in relation to the population (Fig. 2). In this example, there is only one population, i.e. the Labrador retriever. Each graph shows the same data but with a different annotation. Overall, Fig. 2 shows that the sample of interest (dog 1) scores relatively low in terms of heterozygosity, with dog 4 and dog 3 having higher scores (Fig. 2B–D). Different ways of annotating are offered to meet the user’s diverse needs.

The values used to create the graphs, are reported in Table 6.

**Table 6:** Diversity: The level of heterozygosity is calculated by dividing the number of heterozygous loci in the sample of interest by the total number of loci used from the VCF file

ID (dog)	Heterozygosity	Population
1	0.166769630693443	Labrador retriever
2	0.142008520607515	Labrador retriever
3	0.181607682988471	Labrador retriever
4	0.173358258650984	Labrador retriever

Overall, based on the output, a phenotypically healthy, yellow, female Labrador retriever was sequenced. This animal does not require diagnosis or early treatment. However, it did reveal that the animal is a carrier of the variant associated with SD2. This information is important for breeding. In more detail, without excluding the animal for breeding purposes, one can combine this animal with another animal that is wild type homozygous for the variant, responsible for SD2. With this advice, the risk of the condition is limited in the offspring and as few as possible animals are excluded for breeding, contributing to an improved genetic diversity. The worked example thus demonstrates that the variantscanR package allows the analysis of WES data in clinical settings, aids diagnostics and breeding practices. While not applicable in our example, early detection of variants associated with disease might even improve the quality of life if early treatments are available. This is especially the case for diseases with a late onset [34, 35].

### Comparison with other software

In a search for tools with similar goals, we started by defining the characteristics a software tool has to have to solve the practical question we deal with here (see Table 1). Our tool was developed to enable a widespread use of WES/WGS data in veterinary clinical genetics. This led to the following search criteria: “variant filtering” AND “VCF” and “standardized reporting” AND “animal” AND “genetic diversity”. A PubMed search (advanced search: text word and title/abstract) using all these terms returned zero results. Subsampling all possible combinations only started to return results when two main criteria remained. A manual check of these results, returned 11 different tools. These tools are listed in Additional file 5.

VariantscanR is the only existing tool to this day that fulfils criterion #1, i.e. it is capable to identify already known phenotype-associated variants in a VCF file, based on a list provided by the user. All of the 11 tools should be able to handle VCF files (criterion #2). Four of the 11 tools claim to be designed for the use in clinical genetics, of which only 1 tool mentions standardized reporting that can directly be used in this setting (criterion #3). Only 2 tools specifically mention a direct capability to analyze non-human data (criterion #4). None of the tools mentions the option to calculate any measure of genetic diversity (additional criterion #1). In short, variantscanR seems to be the only tool checking all the boxes. This is logical as 9 out of 11 tools were specifically designed to identify new putative phenotype-associated variants based on inheritance assumptions/segregation patterns and 2 other tools focus on appropriately performing variant calling as an alternative to Variant Quality Score Recalibration (VQSR) or hard filtering in the variant calling pipeline.

During the reviewing process, two other tools, that did not come up in the PubMed search, were brought to our attention, i.e. VCF Tools and Oncotator [36, 37]. Since the functionality of both tools partly overlaps with that of variantscanR , we found it useful to cite the differences and individual strengths. The overall goal of filtering in VCF Tools and variantscanR differs. VCF Tools primarily serves as a tool for exploring and manipulating VCF files, whereas variantscanR is specifically designed for filtering known phenotype-associated variants in clinical settings. VariantscanR focuses on screening the VCF file starting with the variants of interest, while VCF Tools begins with the VCF file itself to identify potentially interesting variants with unknown relevance. Consequently, VCF Tools only fulfills the criteria of handling VCF files and allowing non-human samples (i.e. criteria #2 and #4).

The most significant difference between Oncotator and variantscanR is their respective focus. Oncotator is centered around genetic point mutations and short indels in a cancer context, while variantscanR concentrates on germline variants. Although Oncotator was primarily developed for human cancer data analysis, it can be applied to a certain extent for other species. However, it may lack specialized annotations or databases for non-human or non-cancer variants. Oncotator fulfills criteria #1, #2, and #4.

The variantscanR package is dedicated to known phenotype-associated variants in a clinical diagnostic setting, with as the primary goal clear and straightforward reporting as Whole Genome- and Exome sequencing becomes integrated as a routine diagnostic tool in veterinary medicine. It provides concise breeding advice, including information on other samples in the VCF that can be used to identify suitable mating partners. Additionally, the package offers genetic diversity information, and addresses as such one of the prominent challenges in veterinary medicine.

While there may be some overlap in functionalities among existing and future tools, it is crucial to select the most suitable tool for the specific analysis at hand. If the objective is to utilize a user-friendly and straightforward tool that effectively addresses the significant challenges in veterinary medicine, variantscanR comes highly recommended.

As none of the available tools have the same goal, a direct comparison of processing time is not possible. As such, we provide only an overview of the dataset analysed here. The VCF file was 100.854.348 bytes (96.1 MB) big, with 3311 metalines, 13 columns, 4,001,471 gt rows divided over 4 canine samples. On a Windows desktop computer with an Intel(R) core™ i7-8700 CPU @ 3.20 Hhz processor, 16.0 GB installed RAM and a 64-bit operating system × 64-based processor, the total workflow for the worked sample took about 3.8 min (± 232 s). In more detail, the uploading of the VCF file into the R environment and the filtering step were the most time consuming, with a total run time of ± 168 s (2.8 min) and 23 s, respectively. The remaining 3 steps, including the changing of the chromosome notations, the creation of the annotation file and the processing of the VOI file had a duration of respectively, 10, 23 and 8 s.

## Conclusions

Just as in human medicine, high throughput sequencing techniques have the potential to revolutionize routine diagnostics in domestic animals as costs decrease and genetic information becomes more abundant. However, the implementation of these techniques in veterinary diagnostics is hindered by a lack of bioinformatics knowledge among end users.

To address this challenge, our R-package provides user-friendly tools and a standardized analysis framework specifically tailored for genetic and diagnostic laboratories. These laboratories, equipped to offer advanced techniques like whole genome sequencing (WGS) and whole exome sequencing (WES), can utilize our package to generate standardized reports that can be shared with treating veterinarians and breeders. This ensures consistent and effective communication of findings and results.

Other tools often only allow the analysis of human data and/or are not designed to specifically look for a list of variants that are already known to be disease-associated. Capable of analyzing datasets in minutes on standard computers, with only very basic input files that are normally provided by the sequencing facility (a VCF file) or can simply be downloaded (VOI file and annotation file) and processing them in only two steps, variantscanR ticks all the boxes to ensure a widespread implementation. Furthermore, variantscanR can work with data from every species and combines standardized reporting, breeding advice and genetic diversity calculations, making it a unique tool tailored for veterinary clinical genetics.

Ultimately, our aim is to make advanced sequencing techniques more accessible and their implementation feasible. By streamlining the process and providing user-friendly interfaces, our package reduces the need for external high-level bioinformatic support. Over time, this integration enhances the services offered and leverages the power of genomic data analysis.

## Supplementary Information

**Additional file 1:** VariantscanR package vignette. Description of the data: The package vignette serves as a comprehensive guide to help the users understand the package workflow and is provided with worked examples and in-depth instructions on how to use the package functions. The vignette includes code snippets and a step-by-step explanation, demonstrating the package functionality.

**Additional file 2:** Variants called in other samples. Description of data: Variants called in other samples of the multi-VCF file that are present in a wildtype homozygous state in the sample of interest.

**Additional file 3:** Complete output of the variantscanR report for the 4 canine samples. Description of the data: Excel file containing the complete variantscanR output report, including 3 tables per sample analysed. Each sample is displayed on a different excel tab. First table: Priority list of variants that were found in the sample of interest and that are known to segregate in the breed (Labrador retriever) with an established genotype–phenotype association. Second table: Variants in the sample of interest, but not known to segregate in the breed (Labrador retriever). This warrants additional careful interpretation as the genotype–phenotype association can be breed-specific. Third table: Variants called in other samples of the multi-VCF file that are present in a wildtype homozygous state in the sample of interest.

**Additional file 4:** File attributes provided by vcfscanneR function. Original lay out was preserved. Description of data: VCF file attributes provided by the vcfscanneR function after uploading of the VCF file into the R environment. Original lay-out is preserved.

**Additional file 5:** Comparison with other tools. Description of data: A comparison between all tools found in a Pubmed search for the following 4 search terms: VCF, variant filtering, standardised reporting and animal.

## Data Availability

The vignette and accompanying example datasets are made available in the https://github.com/FrederiqueBoeykens/variantscanR. The R script used for the figures and experiments reported in this manuscript is available on https://github.com/FrederiqueBoeykens/variantscanR_extra. The complete VCF file of the 4 canine samples used in this manuscript is available at the European Variation Archive (EVA) database (Accession number: PRJEB63330).

## Abbreviations

NGS: Next Generation Sequencing
WES: Whole Exome Sequencing
WGS: Whole Genome Sequencing
VCF: Variant call format
VOI: Variants of interest
OMIA: Online Mendelian inheritance in Animals
VQSR: Variant Quality Score Recalibration
SD2: Skeletal dysplasia 2
