# Supplementary material for: VariantscanR: an R-package as a clinical tool for variant filtering of known phenotype-associated variants in domestic animals
Source: BMC Bioinformatics. 2023 Aug 1;24:305. doi: 10.1186/s12859-023-05426-6 (PMC10394849; doi:10.1186/s12859-023-05426-6)
Supplement: Supplementary file 1 — Additional file 1: VariantscanR package vignette. Description of the data: The package vignette serves as a comprehensive guide to help the users understand the package workflow and is provided with worked examples and in-depth instructions on how to use the package functions. The vignette includes code snippets and a step-by-step explanation, demonstrating the package functionality. [file 12859_2023_5426_MOESM1_ESM.pdf]

variantscanR vignette

Fréderique Boeykens

2023-07-17

Generic information

Package: variantscanR

Type: Package

Title: Clinical Bioinformatics Tool for Variant Filtering of Known Disease-Causing Mutations in Domestic Animals

Version: 0.0.1 Authors@R: c(person("Fréderique", "Boeykens", email = c("frederique.boeykens@ugent.be", "frederique.boeykens@gmail.com"), role = c("cre", "aut")),person("Bart", "Broeckx", email = "bart.broeckx@ugent.be", role = "aut")) Maintainer: Fréderique Boeykens frederique.boeykens@ugent.be

Description: The R-package variantscanR enables the filtering of variant call format (VCF file) files for the presence of known disease-causing variants. In addition to the main variant-filtering function, the package allows for the estimation of genetic diversity using both single and multisample VCF files. User-defined, file-specific options are available for the quality control of certain parameters using online database screening. Finally, next to the filtering of known disease-causing variants, an additional step is optional to gather the remaining variants located within the genes of interest. The R package is not species-specific.

License: GPL-3

URL: https://github.com/FrederiqueBoeykens/variantscanR``

Encoding: UTF-8

LazyData: True

LazyDataCompression: bzip2

RoxygenNote: 7.1.2

Imports: vcfR, dplyr, stats, rebus, stringr, Biostrings, ggplot2, ggrepel, R3port, svMisc

Suggests: rmarkdown, knitr, BSgenome, BSgenome.Cfamiliaris.UCSC.canFam3, tibble

VignetteBuilder: knitr

Depends: R (>= 2.10)

Installation

Introduction

Since the introduction of next-generation sequencing (NGS) techniques, whole-exome sequencing (WES) and whole-genome sequencing (WGS) not only revolutionized research, but also diagnostics. At present time, most of the tools available for the evaluation of NGS data are solely applicable for human analysis. On top of this, Veterinary Medicine also faces the additional problem that genetic diversity can be dangerously low, especially in the dog. For this reason, we developed *variantscanR*, an *R-package*, for the easy and straightforward identification of known disease-causing variants from a large collection of variants present in dogs and other animals.

The R-package variantscanR enables the filtering of variant call format (VCF file) files for the presence of known disease-causing variants. In addition to the main variant-filtering function, the package allows for the estimation of genetic diversity using both single and multisample VCF files. User-defined, file format-specific options are available for the quality control of certain parameters using online database screening. Finally, next to the filtering of known disease-causing variants, an additional step is optional to gather the remaining variants located within the genes of interest.

VariantscanR is an R package, designed for the use as a clinical tool for variant filtering of known disease-causing variants in domestic animals. The package focuses on cats and dogs but can be used for other animals if the required data and information for this animal is available.

This vignette is divided into four parts:

- Preprocessing
- Variant filtering
- Diversity
- Extra

A visual representation of the workflow is given below. To explain the workflow of this package, an example will be used and elaborated in this vignette. Disclaimer: Not the entire VCF dataset is used for as this is a very large file.

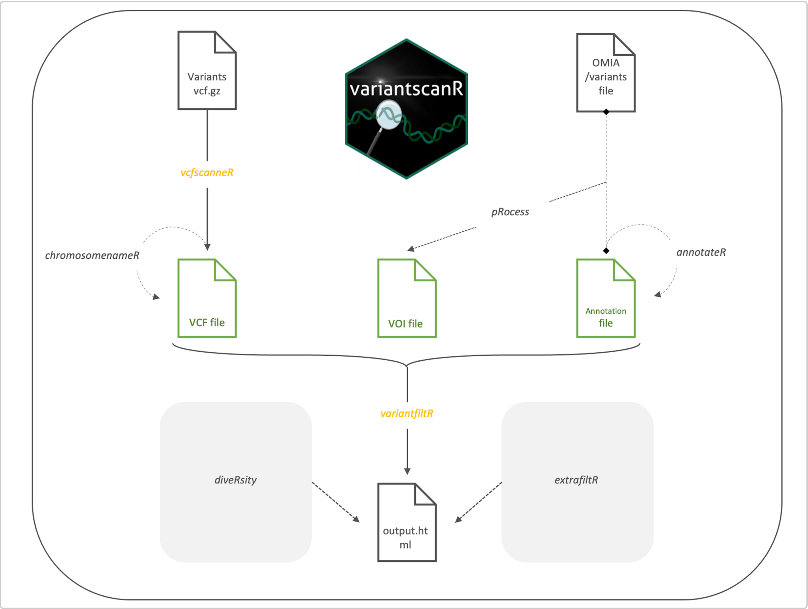

Example: Labrador retriever

WES data were obtained from 16 different dogs for an independent project at the laboratory. The data used in this vignette is for purely demonstrative purposes. One sample, from here on referred to as sample_9, was chosen randomly out of the pool of 16 dogs and is a yellow, female Labrador retriever.

Preprocessing

For the filtering of variants, the pipeline needs a least 3 different input files. The first one being the file containing the variants of interest (VOI file). Secondly, the VCF file to which the variants of interest are filtered against and thirdly, a BED file for the annotation of variants is needed throughout the worflow. These 3 files require a certain format, which is explained in the following sections.

Input files

(1) VCF file

VCF stands for 'Variant Call Format' and is a standardized text file format used for reporting SNPs, indel and structural variations. A VCF file is composed of 3 main parts:

- Meta-data, starting with '##'
- A header that starts with '#CHROM'
- Variant call records

The first 9 columns of the header line and the variant call record line give information on the variants found.

The VCF file already has a certain fixed format and doesn't need adjusting. In fact, please **never edit** a VCF in a word processor by hand, because it **will** disrupt the formatting!

(2) Variants Of Interest file (VOI file)

The VOI file contains the known disease-causing variants you are interested in. This file does need a certain format so it can be used in downstream analysis. Following columns need to be specified:

- **Chromosome**
- **Start**
- End
- Reference
- Reference sequence
- Inheritance pattern
- **Gene**
- Variant Phenotype
- Breed

The columns in **bold** are mandatory, the other ones are optional. The optional columns **need** to be included in the file, but don't need values, so *NA* values are allowed.

However, the function also allows another 'specific' format that can be downloaded straight from the internet, to make it a little more user-friendly. The file can be downloaded from here. For example, if you want to download the known-disease causing variants of the dog, a CSV document can be obtained from here at the bottom **'Download table as CSV'**. You will notice that the *Start*, *End* and *Reference sequence* columns are replaced by a *'g. or m.'* column. The package allows this format. However, make sure adjust the OMIA parameter in the *pRocess* function (explained below) to TRUE (default = FALSE).

You can upload the omia.csv file into the R environment using: file <- read.csv("file_name.csv"). Make sure that the file is located within your working directory, otherwise you can use: file_path <- "path/to/your/file.csv" data <- read.csv(file_path) the *pRocess* function is able to handle the .csv file.

(3) Annotation file

A BED (Browser Extensible Data) file is needed for annotation along the way. A BED file had a fixed format, including 3 required fields:

- chrom
- chromStart
- chromEnd

and 9 additional optional BED fields:

- name
- score
- strand
- thickStart
- thickEnd
- itemRgb
- blockCount
- blockSizes
- blockStarts

Such a BED file can be downloaded here at **the UCSC table Browser**. For our example, we need a BED file for the dog. In our example following criteria were chosen:

- clade: Mammal
- genome: Dog
- assembly: Sep. 2011 (Broad CanFam3.1/canFam3) (*)
- group: Genes and Gene predictions
- track: NCBI RefSeq (*)
- table: RefSeq All (ncbiRefSeq)
- region: genome
- output format: BED - browser extensible data
- output filename: dog_BED_NCBI (*)

(*) These criteria were specifically chosen for this example. Assembly versions change over time and obviously are updated. The assembly you choose should be the same version as the assembly used to create/map the variants in your variants file. Next to *NCBI RefSeq*, you can also choose *Ensembl Genes*. The output file can be found in the package Data folder.

However, this BED file itself needs some extra annotation. For this reason yet another file needs to be downloaded from the same site. The same criteria apply for this file as the previous BED file, except for the output format. Instead of chosing *BED - browser extensible data*, *all fields from selected table* is required.

And that was it! Now you are ready to do some variant filtering!

(Extra) Chromosome names table

Next, depending on the reference genomes used for creating the VCF file, the chromosome notation can have different denominations. The *chromosomenameR* function allows the conversion, if needed. The chromosomes have multiple different nomenclatures depending on the reference used for mapping. For example, in dogs, chromosome 1 can be noted as CM000001.3 or NC_006583.3, using the GenBank or RefSeq sequence, respectively. This notation needs to be changed to the 'chrN' notation, with N being the number of the chromosome. This is done using the *chromosomerenamR* function.

For this conversion a file a needed providing both names. For the dog, such a file is available in the package Data folder. The original names are stored in the **first** column and the 'chrN' names are placed in the **second** column.

VCFscanneR

Because the VCF file format is not one of the standard formats that can be uploaded in the R environment, the vcfscannR function was created. This function uploads a multisample sample VCF file into the R environment and turns it into a single sample VCF, ready for downstream analysis. The function has 2 parameters. The first parameter is the name of the VCF file that needs to be uploaded OR contains the path to the file. Both .vcf and .vcf.gz formats are accepted. The second parameter is the name of the sample of interest. **The sample name has to match with that of the VCF file**. In our example, the name is *sample_9*.

The example VCF file (*SNPs.recode.subset.rename.vcf.gz*) has 31 rows and 10 columns and contains variants found within chromosome 5.

After uploading the multisample VCF file with *vcfscanneR* function, the VCF looks like this:

```
require(dplyr)
library(variantscanR)
pkg <- "variantscanR"
vcf_file <- system.file("extdata", "SNPs.recode.subset.rename.vcf.gz", package = pkg)
sample <- "sample_9"
vcf <- vcfscanneR(vcf_file, sample)
```

```
## Scanning file to determine attributes.
## File attributes:
##   meta lines: 3311
##   header_line: 3312
##   variant count: 31
##   column count: 36
##
Meta line 1000 read in.
Meta line 2000 read in.
```

```
Meta line 3000 read in.
Meta line 3311 read in.
## All meta lines processed.
## gt matrix initialized.
## Character matrix gt created.
##   Character matrix gt rows: 31
##   Character matrix gt cols: 36
##   skip: 0
##   nrows: 31
##   row_num: 0
##
Processed variant: 31
## All variants processed
```

```
head(vcf)
```

```
##         chrom      pos   id ref alt    qual filter info                 format
## 1 NC_006587.3 63684323 <NA>   A   C 1562.62   PASS <NA>         GT:AD:DP:GQ:PL
## 2 NC_006587.3 63684670 <NA>   A   T 19640.7   PASS <NA> GT:AD:DP:GQ:PGT:PID:PL
## 3 NC_006587.3 63684965 <NA>   C   T   80.71   PASS <NA>         GT:AD:DP:GQ:PL
## 4 NC_006587.3 63685053 <NA>   G   A  544.68   PASS <NA>         GT:AD:DP:GQ:PL
## 5 NC_006587.3 63685230 <NA>   G   A    61.6   PASS <NA>         GT:AD:DP:GQ:PL
## 6 NC_006587.3 63685760 <NA>   A   G 1893.16   PASS <NA> GT:AD:DP:GQ:PGT:PID:PL
##                       sample
## 1       0/1:5,4:9:99:140,0,140
## 2 1/1:0,31:31:93:.:.:1242,93,0
## 3        0/0:6,0:6:15:0,15,225
## 4            ./.:2,0:2:.:0,0,0
## 5            ./.:0,0:0:.:0,0,0
## 6        ./.:3,0:3:.:.:.:0,0,0
```

As you can see, the chromosomes do not have the required 'chrN' notation, for this the *chromosomenameR* function needs to be used.

chromosomenameR

```
a <- vcf
b <- dog_chromosome_names
vcf_chr <- chromosomenameR(a,b)
head(vcf_chr)
```

```
##   chrom      pos   id ref alt    qual filter info                 format
## 1  chr5 63684323 <NA>   A   C 1562.62   PASS <NA>         GT:AD:DP:GQ:PL
## 2  chr5 63684670 <NA>   A   T 19640.7   PASS <NA> GT:AD:DP:GQ:PGT:PID:PL
## 3  chr5 63684965 <NA>   C   T   80.71   PASS <NA>         GT:AD:DP:GQ:PL
## 4  chr5 63685053 <NA>   G   A  544.68   PASS <NA>         GT:AD:DP:GQ:PL
## 5  chr5 63685230 <NA>   G   A    61.6   PASS <NA>         GT:AD:DP:GQ:PL
## 6  chr5 63685760 <NA>   A   G 1893.16   PASS <NA> GT:AD:DP:GQ:PGT:PID:PL
##                       sample
## 1       0/1:5,4:9:99:140,0,140
## 2 1/1:0,31:31:93:.:.:1242,93,0
## 3        0/0:6,0:6:15:0,15,225
## 4            ./.:2,0:2:.:0,0,0
## 5            ./.:0,0:0:.:0,0,0
## 6        ./.:3,0:3:.:.:.:0,0,0
```

Now, our VCF file is ready for variant filtering.

annotateR_NCBI.R

As was explained in the input section above, the BED file itself needs anotating. For this step, 2 separate functions were created that essentially do the same thing. One function can be used for a *NCBI RefSeq* BED file. Whereas the other function was designed to handle an *Ensemble Genes* (see section 'input file').

For demonstrative purposes, only the NCBI specific function, *annotateR_NCBI* function will be demonstrated for as the BED file used in this example is *NCBI RefSeq* based.

This is what the NCBI based BED file looks like:

```
data("dog_BED_NCBI", package = "variantscanR")
head(dog_BED_NCBI)
```

```
##     V1       V2       V3             V4 V5 V6       V7       V8 V9 V10
## 1 chr1 67080313 67123622 XR_001316235.2  0  + 67123622 67123622  0   5
## 2 chr1  8348627  8437520 XM_022413126.1  0  +  8351206  8435145  0   7
## 3 chr1  8348627  8437520 XM_005615331.3  0  +  8351206  8435145  0   7
## 4 chr1  8358489  8437520 XM_022413174.1  0  +  8365670  8435145  0   7
## 5 chr1 16597388 16819888    XM_847492.3  0  + 16597388 16819782  0  10
```

```
## 6 chr1 49886886 51201930    XM_850059.5  0  - 49889004 51201764  0  12
##                                             V11
## 1                           365,71,169,96,1439,
## 2                  2592,72,339,348,103,52,2489,
## 3                  2592,72,339,360,103,52,2489,
## 4                   958,72,339,360,103,52,2489,
## 5         218,53,135,153,104,121,140,36,36,340,
## 6 2231,118,84,150,62,137,116,84,122,238,164,173,
##                                                                         V12
## 1                                                    0,670,8419,39776,41870,
## 2                                     0,17017,17396,61752,79246,83425,86404,
## 3                                     0,17017,17396,61752,79246,83425,86404,
## 4                                       0,7155,7534,51890,69384,73563,76542,
## 5             0,193006,197159,199502,209046,214934,220292,220531,221245,222160,
## 6 0,7480,40500,189511,206105,416390,580991,668985,818897,880229,1049798,1314871,
```

You can see that the 3 required and the 9 additional fields are present. Now let's annotate this file with the second file we downloaded from UCSC table browser.

```
data("dog_BED_NCBI", package = "variantscanR")
data("dog_allfields_NCBI", package = "variantscanR")
a <- dog_BED_NCBI
b <- dog_allfields_NCBI
BED_file_fully_annotated <- annotateR_NCBI(a,b)
head(BED_file_fully_annotated)
```

```
##     V1       V2       V3           V4 V5 V6       V7       V8 V9 V10
## 1 chr1 67080313 67123622 LOC106558822  0  + 67123622 67123622  0   5
## 2 chr1  8348627  8437520        CD226  0  +  8351206  8435145  0   7
## 3 chr1  8348627  8437520        CD226  0  +  8351206  8435145  0   7
## 4 chr1  8358489  8437520        CD226  0  +  8365670  8435145  0   7
## 5 chr1 16597388 16819888        CCBE1  0  + 16597388 16819782  0  10
## 6 chr1 49886886 51201930         PRKN  0  - 49889004 51201764  0  12
##                                             V11
## 1                           365,71,169,96,1439,
## 2                  2592,72,339,348,103,52,2489,
## 3                  2592,72,339,360,103,52,2489,
## 4                   958,72,339,360,103,52,2489,
## 5         218,53,135,153,104,121,140,36,36,340,
## 6 2231,118,84,150,62,137,116,84,122,238,164,173,
##                                                                         V12
## 1                                                    0,670,8419,39776,41870,
## 2                                     0,17017,17396,61752,79246,83425,86404,
## 3                                     0,17017,17396,61752,79246,83425,86404,
## 4                                       0,7155,7534,51890,69384,73563,76542,
## 5             0,193006,197159,199502,209046,214934,220292,220531,221245,222160,
## 6 0,7480,40500,189511,206105,416390,580991,668985,818897,880229,1049798,1314871,
##              V13
## 1 XR_001316235.2
## 2 XM_022413126.1
## 3 XM_005615331.3
## 4 XM_022413174.1
## 5    XM_847492.3
## 6    XM_850059.5
```

A 13th column (V13) is added, containing the gene names instead of just the transcripts that are present in the BED file with 12 columns. This is important because *the variants file* only contains gene names and not transcript names.

NOTE: The entire workflow has been performed on the same dataset using the same *variants file* for both the *NCBI refseq* BED file and the *Ensembl genes* BED file. It was noticed that overall less variants were attained after filtering with the *Ensembl genes* BED file compared to the *NCBI refseq* BED file. This is mostly due to different annotation of the reference sequence and that less gene names were present/annotated in the *Ensembl genes* BED file.

pRocess

Now that all the input files have been amended for downstream analysis. A final preprocessing step, that is a mandatory in the pipeline, can be performed.

The *pRocess* function has several parameters of which a couple are optional. Because files that are manually made or adjusted are prone to human errors, quality control might be useful. The *pRocess* function includes some optional quality control filters. For example, the genomic locations provided for each variants are screened for errors by comparison with a reference genome. If a mismatch is encountered, the user will get notified of this error and will be asked to check this information for this variant. Next to this, an assembly version check can be performed by providing the "refseq" parameter with this information. If a variant does not belong to the right verion, that variant is removed from the VCF file. This is *"CanFam3.1"* in our example. If the *variants file* is downloaded from the OMIA website (as explained above in the Input files section), it is possible to provide the function with this information by setting the *OMIA paramater* to TRUE (this is FALSE by default).

In summary, we have 6 parameters:

1. variants_file: *Required*

2. BED_file: *Required*
3. refseq: *Optional* and is TRUE by default
4. QC: *Optional*
5. organism: *Optional* but *Required* if QC = TRUE
6. OMIA: *Optional* and is FALSE by default

Test 1 pRocess parameters: Quality control: yes, OMIA = TRUE and QC = TRUE

```
data("final_omia_dataset", package = "variantscanR")
variants_file <- final_omia_dataset
BED_file <- BED_file_fully_annotated
refseq <- "CanFam3.1"
organism <- BSgenome.Cfamiliaris.UCSC.canFam3
variant_file_processed_1 <- pRocess(variants_file, BED_file, refseq, QC = TRUE, organism, OMIA
  = TRUE)
head(variant_file_processed_1)
```

```
##   Chromosome    Start      End Reference          Check orientation
## 1       chr6 55146556       NA           Check manually           +
## 2       chr5 32193689       NA         C              C           +
## 3      chr20 53101896       NA         C Check manually           -
## 4       chr3 40614853 40614872           Check manually           +
## 5       chr3 40782144       NA         G              G           +
## 6       chr3 40808345       NA         G              G           +
##   Reference.Sequence Inheritance.pattern     Gene
## 1          CanFam3.1                  NA    ABCA4
## 2          CanFam3.1                  NA   ACADVL
## 3          CanFam3.1                  NA ADAMTS10
## 4          CanFam3.1                  NA ADAMTS17
## 5          CanFam3.1                  NA ADAMTS17
## 6          CanFam3.1                  NA ADAMTS17
##                              Variant.Phenotype
## 1                            Stargardt disease 1
## 2            Exercise induced metabolic myopathy
## 3                   Glaucoma, primary open angle
## 4 Glaucoma, primary open angle, ADAMTS17-related
## 5                                 Lens luxation
## 6 Glaucoma, primary open angle, ADAMTS17-related
##
Breed
## 1
Labrador Retriever
## 2
German Hunting Terrier
## 3
Norwegian Elkhound
## 4
Basset Hound
## 5 Chinese Crested Dog, German Hunting Terrier, Jack Russell Terrier, Lancashire heeler,
Miniature Bull Terrier, Parson Russell Terrier, Patterdale Terrier, Rat Terrier, Sealyham
Terrier, Tenterfield Terrier, Tibetan Terrier, Toy Fox Terrier, Volpino Italiano, Welsh
Terrier, Wirehaired Fox Terrier, Yorkshire Terrier
## 6
Basset Fauve de Bretagne
```

This shows a good example of the quality control that is performed on the location of the variants. For the first SNP located on chromosome 6 at location 55146549, the user-defined *variants_file* states that a C is should be present at that location. However, when comparing with an online Reference Sequence, no match is found. The output tells the user to double check the information on this variant. When looking up this variant on OMIA.org, from where the file was downloaded, it shows that it is an insertion of C on that location, which explains why it did not match the reference. For as all the other information was correct, it won't cause any problems for downstream analysis. For the 5th variant, no reference was given and therefore, that row needs revisioning.

test 2 pRocess paramaters: Quality control: No, OMIA = TRUE and QC = FALSE

```
data("final_omia_dataset", package = "variantscanR")
variants_file <- final_omia_dataset
BED_file <- BED_file_fully_annotated
refseq <- "CanFam3.1"
organism <- BSgenome.Cfamiliaris.UCSC.canFam3
variant_file_processed <- pRocess(variants_file, BED_file, refseq, QC = FALSE, organism, OMIA =
  TRUE)
head(variant_file_processed)
```

```
##   Chromosome    Start      End Reference Reference.Sequence Inheritance.pattern
## 1       chr6 55146556     <NA>                     CanFam3.1                <NA>
## 2       chr5 32193689     <NA>         C          CanFam3.1                <NA>
## 3      chr20 53101896     <NA>         C          CanFam3.1                <NA>
## 4       chr3 40614853 40614872                    CanFam3.1                <NA>
## 5       chr3 40782144     <NA>         G          CanFam3.1                <NA>
## 6       chr3 40808345     <NA>         G          CanFam3.1                <NA>
```

```
##       Gene                                Variant.Phenotype
## 1    ABCA4                              Stargardt disease 1
## 2   ACADVL             Exercise induced metabolic myopathy
## 3 ADAMTS10                     Glaucoma, primary open angle
## 4 ADAMTS17 Glaucoma, primary open angle, ADAMTS17-related
## 5 ADAMTS17                                    Lens luxation
## 6 ADAMTS17 Glaucoma, primary open angle, ADAMTS17-related
##
Breed
## 1
Labrador Retriever
## 2
German Hunting Terrier
## 3
Norwegian Elkhound
## 4
Basset Hound
## 5 Chinese Crested Dog, German Hunting Terrier, Jack Russell Terrier, Lancashire heeler,
Miniature Bull Terrier, Parson Russell Terrier, Patterdale Terrier, Rat Terrier, Sealyham
Terrier, Tenterfield Terrier, Tibetan Terrier, Toy Fox Terrier, Volpino Italiano, Welsh
Terrier, Wirehaired Fox Terrier, Yorkshire Terrier
## 6
Basset Fauve de Bretagne
```

This test shows the result of a variants file processed by the pRocess function but without the quality control (QC = FALSE). No *check* or *orientation* columns are provided and no warnings are given concerning the location of the variants. This way of processing might come in handy if it is preferred to run the pipeline without using the *BSgenome reference genomes*. If QC = FALSE, the *organism* parameter can be left out.

test 3: Other format provided, OMIA = FALSE, QC = TRUE

```
data("variants_file_alternative", package = "variantscanR")
variants_file <- variants_file_alternative
BED_file <- BED_file_fully_annotated
organism <- BSgenome.Cfamiliaris.UCSC.canFam3
variant_file_processed <- pRocess(variants_file, BED_file, refseq, QC = FALSE, organism, OMIA =
  FALSE)
head(variant_file_processed)
```

```
##   Chromosome    Start      End Reference Reference Sequence Inheritance.pattern
## 1       chr6 55146549     <NA>         C          CanFam3.1                <NA>
## 2       chr5 32193689     <NA>         C          CanFam3.1                <NA>
## 3      chr20 53101896     <NA>         G          CanFam3.1                <NA>
## 4       chr3 40935387 40935392    CGTGGT          CanFam3.1                <NA>
## 5       chr3 40614853 40614872                    CanFam3.1                <NA>
## 6       chr3 40782144     <NA>         G          CanFam3.1                <NA>
##       Gene
## 1    ABCA4
## 2   ACADVL
## 3 ADAMTS10
## 4 ADAMTS17
## 5 ADAMTS17
## 6 ADAMTS17
##                                                        Variant.Phenotype
## 1                                                    Stargardt disease 1
## 2                                    Exercise induced metabolic myopathy
## 3                                           Glaucoma, primary open angle
## 4 Primary open-angle glaucoma (POAG), primary lens luxation (PLL), or both
## 5                         Glaucoma, primary open angle, ADAMTS17-related
## 6                                                          Lens luxation
##                                                         Breed       OMIA.ID.s.
## 1                                          Labrador Retriever OMIA 002179-9615
## 2                                      German Hunting Terrier OMIA 002140-9615
## 3                                          Norwegian Elkhound OMIA 001870-9615
## 4                                            Chinese Shar-Pei OMIA 001976-9615
## 5                                                Basset Hound OMIA 001976-9615
## 6 American hairless, Chinese Crested Dog, Jack Russell Terrier OMIA 000588-9615
##   Species.Name Allele         Type.of.Variant Deleterious.             c..or.n.
## 1          dog   <NA> insertion, small (<=20)          yes           c.4176insC
## 2          dog   <NA>    nonsense (stop-gain)      unknown            c.1728C>A
## 3          dog   <NA>                missense          yes            c.1159G>A
## 4          dog   <NA>  deletion, small (<=20)          yes c.3070_3075delCGTGGT
## 5          dog   <NA>  deletion, small (<=20)          yes                 <NA>
## 6          dog   <NA>                splicing          yes           c.1473+1G>A
##                  p. EVA.ID Year.Published PubMed.ID.s.
## 1      p.F1393Lfs*1395   <NA>           2019     30889179
## 2            p.Tyr576*   <NA>           2018     29491033
## 3              p.A387T   <NA>           2014     25372548
## 4 p.Val1025_Val1026del   <NA>           2018     29287154
## 5                 <NA>   <NA>           2015     26474315
## 6                 <NA>   <NA>           2010     20375329
```

Variant filtering

After the preprocessing step are done, the filtering step can be performed.

variantfiltR

```
require(stringr)
vcf <- vcf_chr
variants_file <- variant_file_processed_1
BED_file_annot <- BED_file_fully_annotated
breed <- "Labrador retriever"
report <- variantfiltR(vcf, variants_file, BED_file_annot, breed)
```

```
## Progress:   1 on 161  Progress:   2 on 161  Progress:   3 on 161  Progress:   4 on 161
Progress:   5 on 161  Progress:   6 on 161  Progress:   7 on 161  Progress:   8 on 161
Progress:   9 on 161  Progress:  10 on 161  Progress:  11 on 161  Progress:  12 on 161
Progress:  13 on 161  Progress:  14 on 161  Progress:  15 on 161  Progress:  16 on 161
Progress:  17 on 161  Progress:  18 on 161  Progress:  19 on 161  Progress:  20 on 161
Progress:  21 on 161  Progress:  22 on 161  Progress:  23 on 161  Progress:  24 on 161
Progress:  25 on 161  Progress:  26 on 161  Progress:  27 on 161  Progress:  28 on 161
Progress:  29 on 161  Progress:  30 on 161  Progress:  31 on 161  Progress:  32 on 161
Progress:  33 on 161  Progress:  34 on 161  Progress:  35 on 161  Progress:  36 on 161
Progress:  37 on 161  Progress:  38 on 161  Progress:  39 on 161  Progress:  40 on 161
Progress:  41 on 161  Progress:  42 on 161  Progress:  43 on 161  Progress:  44 on 161
Progress:  45 on 161  Progress:  46 on 161  Progress:  47 on 161  Progress:  48 on 161
Progress:  49 on 161  Progress:  50 on 161  Progress:  51 on 161  Progress:  52 on 161
Progress:  53 on 161  Progress:  54 on 161  Progress:  55 on 161  Progress:  56 on 161
Progress:  57 on 161  Progress:  58 on 161  Progress:  59 on 161  Progress:  60 on 161
Progress:  61 on 161  Progress:  62 on 161  Progress:  63 on 161  Progress:  64 on 161
Progress:  65 on 161  Progress:  66 on 161  Progress:  67 on 161  Progress:  68 on 161
Progress:  69 on 161  Progress:  70 on 161  Progress:  71 on 161  Progress:  72 on 161
Progress:  73 on 161  Progress:  74 on 161  Progress:  75 on 161  Progress:  76 on 161
Progress:  77 on 161  Progress:  78 on 161  Progress:  79 on 161  Progress:  80 on 161
Progress:  81 on 161  Progress:  82 on 161  Progress:  83 on 161  Progress:  84 on 161
Progress:  85 on 161  Progress:  86 on 161  Progress:  87 on 161  Progress:  88 on 161
Progress:  89 on 161  Progress:  90 on 161  Progress:  91 on 161  Progress:  92 on 161
Progress:  93 on 161  Progress:  94 on 161  Progress:  95 on 161  Progress:  96 on 161
Progress:  97 on 161  Progress:  98 on 161  Progress:  99 on 161  Progress: 100 on 161
Progress: 101 on 161  Progress: 102 on 161  Progress: 103 on 161  Progress: 104 on 161
Progress: 105 on 161  Progress: 106 on 161  Progress: 107 on 161  Progress: 108 on 161
Progress: 109 on 161  Progress: 110 on 161  Progress: 111 on 161  Progress: 112 on 161
Progress: 113 on 161  Progress: 114 on 161  Progress: 115 on 161  Progress: 116 on 161
Progress: 117 on 161  Progress: 118 on 161  Progress: 119 on 161  Progress: 120 on 161
Progress: 121 on 161  Progress: 122 on 161  Progress: 123 on 161  Progress: 124 on 161
Progress: 125 on 161  Progress: 126 on 161  Progress: 127 on 161  Progress: 128 on 161
Progress: 129 on 161  Progress: 130 on 161  Progress: 131 on 161  Progress: 132 on 161
Progress: 133 on 161  Progress: 134 on 161  Progress: 135 on 161  Progress: 136 on 161
Progress: 137 on 161  Progress: 138 on 161  Progress: 139 on 161  Progress: 140 on 161
Progress: 141 on 161  Progress: 142 on 161  Progress: 143 on 161  Progress: 144 on 161
Progress: 145 on 161  Progress: 146 on 161  Progress: 147 on 161  Progress: 148 on 161
Progress: 149 on 161  Progress: 150 on 161  Progress: 151 on 161  Progress: 152 on 161
Progress: 153 on 161  Progress: 154 on 161  Progress: 155 on 161  Progress: 156 on 161
Progress: 157 on 161  Progress: 158 on 161  Progress: 159 on 161  Progress: 160 on 161
Progress: 161 on 161  Progress: 1 on 2  Progress: 2 on 2  Progress: 1 on 2  Progress: 2 on 2
Progress: 1 on 2  Progress: 2 on 2  Progress: 1 on 2  Progress: 2 on 2  Progress: 1 on 2
Progress: 2 on 2
```

```
head(report)
```

```
##   Chromosome Location Gene Exon.or.Intron Wild.Type Allele.1 Allele.2
## 1   Variants  present   in         sample       and    found       in
## 2
## 3       chr5 63694334 MC1R       Intronic         G        A        A
## 4
## 5   Variants  present   in         sample       but      not    found
## 6
##     Zygosity Refseq.Transcript Inheritance.Pattern Variant.Phenotype
## 1      breed                of            interest                 !
## 2
## 3 Homozygous    NM_001014282.2                  NA   Red/yellow coat
## 4
## 5         in             breed                  of          interest
## 6
##                          Breed.s.
## 1                               :
## 2
## 3 Irish Setter, Labrador Retriever
```

```
## 4
## 5                                         :
## 6
```

The output of this function is difficult to show because raw .html files are created that will pop up in your internet browser. This head of the report is shown here, but it is not very appealing. For this reason, images of the output are provided in the following section.

Reporting

For demonstrative purposes, not the entire VCF dataset was used for the worked out example in this vignette. However, we do believe it is important to work out a real example using the entire dataset and show the outcome. For this reason, images were made of the real outcome and are shown below.

So if we go through the entire pipeline, after the *variantfiltR* function, an html report is created. The minimal output is an interactive overview of the various table created after filtering (fig2). By clicking on the desired table, the output will be displayed.

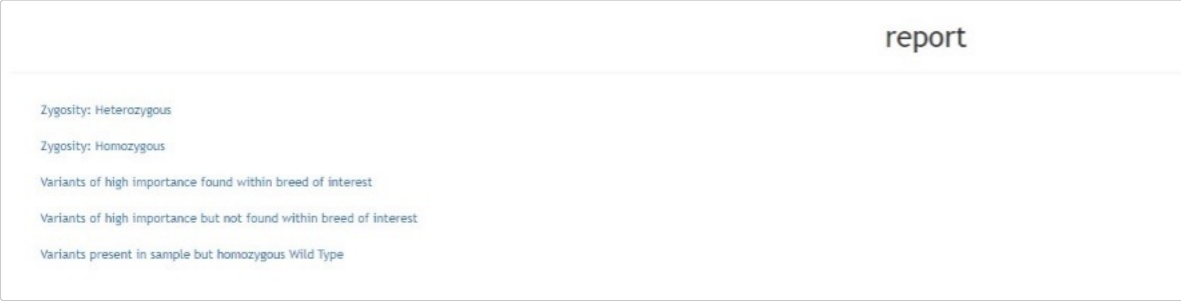

Figure 2: Report overview of tables

Next to this, breeding advice is also provided for heterozygous (fig3) and homozygous (fig4) variants according to the inheritance pattern.

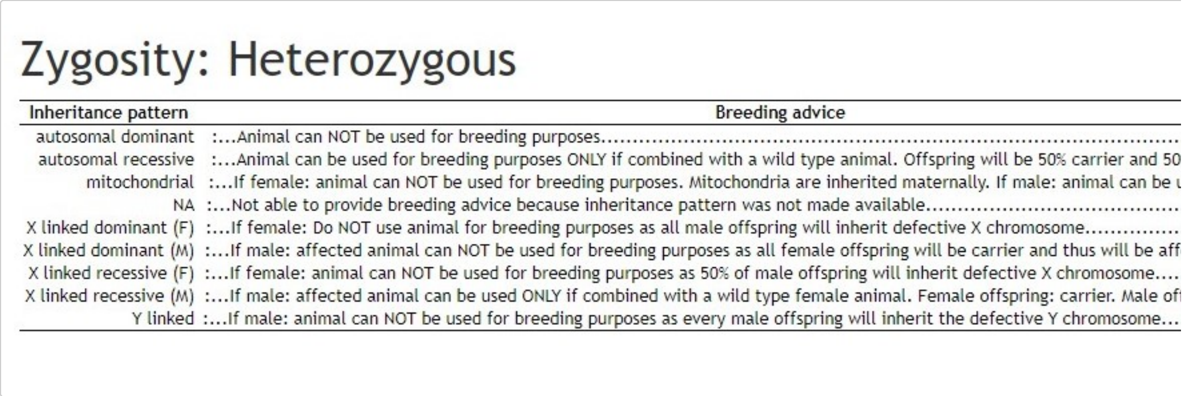

Zygosity: Heterozygous

Inheritance pattern	Breeding advice
autosomal dominant	:…Animal can NOT be used for breeding purposes.……
autosomal recessive	:…Animal can be used for breeding purposes ONLY if combined with a wild type animal. Offspring will be 50% carrier and 50
mitochondrial	:…If female: animal can NOT be used for breeding purposes. Mitochondria are inherited maternally. If male: animal can be u
NA	:…Not able to provide breeding advice because inheritance pattern was not made available.……
X linked dominant (F)	:…If female: Do NOT use animal for breeding purposes as all male offspring will inherit defective X chromosome.……
X linked dominant (M)	:…If male: affected animal can NOT be used for breeding purposes as all female offspring will be carrier and thus will be aff
X linked recessive (F)	:…If female: animal can NOT be used for breeding purposes as 50% of male offspring will inherit defective X chromosome.…
X linked recessive (M)	:…If male: affected animal can be used ONLY if combined with a wild type female animal. Female offspring: carrier. Male off
Y linked	:…If male: animal can NOT be used for breeding purposes as every male offspring will inherit the defective Y chromosome…

Figure 3: Heterozygous breeding advice

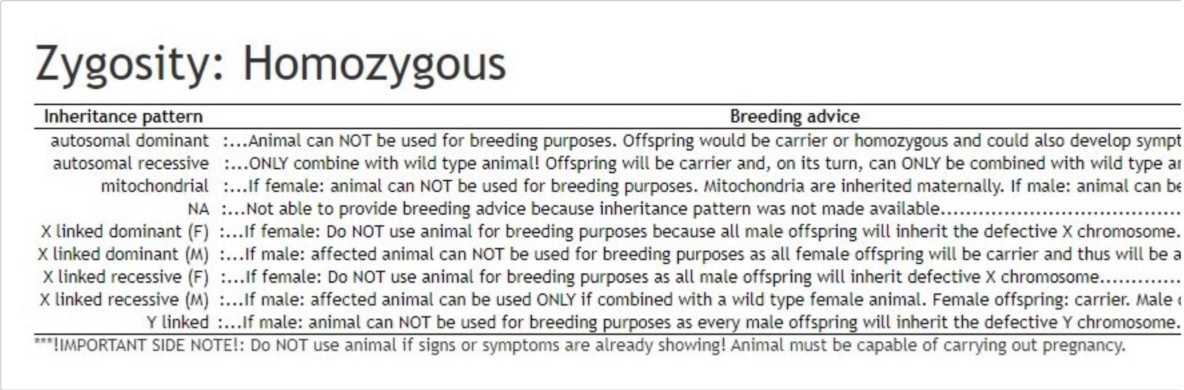

Zygosity: Homozygous

Inheritance pattern	Breeding advice
autosomal dominant	:…Animal can NOT be used for breeding purposes. Offspring would be carrier or homozygous and could also develop sympt
autosomal recessive	:…ONLY combine with wild type animal! Offspring will be carrier and, on its turn, can ONLY be combined with wild type an
mitochondrial	:…If female: animal can NOT be used for breeding purposes. Mitochondria are inherited maternally. If male: animal can be
NA	:…Not able to provide breeding advice because inheritance pattern was not made available.……
X linked dominant (F)	:…If female: Do NOT use animal for breeding purposes because all male offspring will inherit the defective X chromosome.
X linked dominant (M)	:…If male: affected animal can NOT be used for breeding purposes as all female offspring will be carrier and thus will be a
X linked recessive (F)	:…If female: Do NOT use animal for breeding purposes as all male offspring will inherit defective X chromosome.……
X linked recessive (M)	:…If male: affected animal can be used ONLY if combined with a wild type female animal. Female offspring: carrier. Male o
Y linked	:…If male: animal can NOT be used for breeding purposes as every male offspring will inherit the defective Y chromosome.

***!IMPORTANT SIDE NOTE!: Do NOT use animal if signs or symptoms are already showing! Animal must be capable of carrying out pregnancy.

Figure 4: Homozygous breeding advice

Finally, if a breed is provided as a parameter in the *variantfiltR* function, 3 other tables are acquired. One table containes the most important variants, being the variants that are not homozygous wild type AND are found within the breed of interest. Because known-disease causing variants are checked within the animal of interest, these variants might be important for health and diagnostics. (fig5).

Variants of high importance found within breed of interest

Chromosome	Location	Gene	Exon or Intron	Wild Type	Allele 1	Allele 2	Zygosity	Refseq Transcript	Inheritance Pattern	Variant Phe
chr12	2652874	COL11A2	exon60	C	C	G	Heterozygous	XM_005627196.2	autosomal recessive	Skeletal dyspla
								XM_022425927.1	autosomal recessive	Skeletal dyspla
			exon61	C	C	G	Heterozygous	XM_014118359.1	autosomal recessive	Skeletal dyspla
								XM_022425925.1	autosomal recessive	Skeletal dyspla
								XM_022425926.1	autosomal recessive	Skeletal dyspla
			exon62	C	C	G	Heterozygous	XM_014118358.1	autosomal recessive	Skeletal dyspla
								XM_022425923.1	autosomal recessive	Skeletal dyspla
			exon63	C	C	G	Heterozygous	XM_005627195.3	autosomal recessive	Skeletal dyspla
								XM_014118356.1	autosomal recessive	Skeletal dyspla
								XM_022425924.1	autosomal recessive	Skeletal dyspla
								XM_538855.5	autosomal recessive	Skeletal dyspla
			Intronic	C	C	G	Heterozygous	XR_002631768.1	autosomal recessive	Skeletal dyspla
chr5	63694334	MC1R	exon1	G	A	A	Homozygous	NM_001014282.2	autosomal recessive	Red/y
chrX	60279238	ATP7A	exon2	C	C	T	Heterozygous	XM_005641522.2	X linked recessive	Menl
			Intronic	C	C	T	Heterozygous	XM_005641518.2	X linked recessive	Menl
								XM_005641519.2	X linked recessive	Menl
								XM_005641520.2	X linked recessive	Menl
								XM_005641521.2	X linked recessive	Menl
								XM_014111853.2	X linked recessive	Menl
								XM_022415666.1	X linked recessive	Menl
								XM_022415667.1	X linked recessive	Menl
								XM_022415668.1	X linked recessive	Menl
								XM_022415669.1	X linked recessive	Menl
								XM_549096.5	X linked recessive	Menl

Sample: sample_9

Figure 5

The second table contains variants that are not homozygous wild type but are not known to have implications in or known to occur in the breed of interest. Still, this kind of information might be important to report (fig6).

Variants of high importance but not found within breed of interest

Chromosome	Location	Gene	Exon or Intron	Wild Type	Allele 1	Allele 2	Z
chr32	4509367	FGF5	exon1	G	A call cannot be made for this sample at this given locus	A call cannot be made for this sample at this given locus	Zygo
chr5	63694460	MC1R	exon1	C	T	T	

Sample: sample_9

Figure 6

Lastly, the third table contain the variants found or not found within the breed of interest that are homozygous wild type (fig7).

Variants present in sample but homozygous Wild Type

Chromosome	Location	Gene	Exon or Intron	Wild Type	Allele 1	Allele 2	Zygosity	Refseq Transcript	Inheritance Pattern	Variant Phenotype	
chr11	33317810	TYRP1	exon1	T	T	T	Homozygous	NM_001194966.1	NA	Brown	
			Intronic	T	T	T	Homozygous	XM_005626363.2	NA	Brown	
	33326685	TYRP1	exon4	C	C	C	Homozygous	NM_001194966.1	NA	Brown	
			Intronic	C	C	C	Homozygous	XM_005626363.2	NA	Brown	
chr13	61287796	ADAMTS3	Intronic	G	G	G	Homozygous	XM_539311.5	NA	Upper airway syndrome	
chr2	21731842	SUV39H2	exon2	A	A	A	Homozygous	XM_005617110.3	NA	Nasal parakeratosis	
								XM_005617111.2	NA	Nasal parakeratosis	
								XM_005617112.3	NA	Nasal parakeratosis	
								XM_535179.6	NA	Nasal parakeratosis	
			Intronic	A	A	A	Homozygous	XM_005617109.3	NA	Nasal parakeratosis	
								XM_005617114.3	NA	Nasal parakeratosis	
chr20	55850145	MFSD12	Intronic	C	C	C	Homozygous	XM_005633234.1	NA	White or cream	
chr24	23393552	ASIP	Intronic	C	C	C	Homozygous	NM_001007263.1	NA	Recessive black	
								XM_014106843.2	NA	Recessive black	
								XM_022408819.1	NA	Recessive black	
								XM_022408820.1	NA	Recessive black	
chr30	37821686	CYP1A2	exon3	C	C	C	Homozygous	NM_001008720.1	NA	Metabolizer of a cognitive enhancer	
								XM_014109343.2	NA	Metabolizer of a cognitive enhancer	
chr9	4188663	PRCD	Intronic	C	C	C	Homozygous	NM_001097560.2	NA	Progressive rod-cone degeneration	Ame Entl
								XM_022422503.1	NA	Progressive rod-cone degeneration	Ame Entl
	55282762	DNM1	exon17	C	C	C	Homozygous	NM_001131049.1	autosomal recessive	Exercise-induced collapse	
chrM	14474	CYTB	exon1	G	G	G	Homozygous	NP_008483.1	Mitochondrial	Leucodystrophy	

Sample: sample_9

Figure 7

Diversity

The *variantscanR* package also features a diversity analysis.

diveRsity

The input is a multisample VCF file and the name of one specific sample the user is interested in. Next to the VCF file, the user should also provide a file (this can be for example an Excel file) with the breeds of the samples. The order of the rows should match the order of the samples in the VCF file.This means that the first row contains the breed of the first sample and so on. Please make sure that the column name for that column is 'Breed' with the capital letter 'B'.

This shows the first 6 rows of an example 'Breeds' file:

```
data("breeds", package = "variantscanR")
breeds <- breeds
head(breeds)
```

```
## # A tibble: 6 × 1
##   Breed
##   <chr>
## 1 Labrador retriever
## 2 Labrador retriever
## 3 Labrador retriever
## 4 Labrador retriever
## 5 Labrador retriever
## 6 Labrador retriever
```

You can upload an Excel file into the R environment like this:

Breeds <- read_excel("path_to_your_file.xlsx")

Changing the column names can be done like this:

colnames(Breeds) <- "Breed"

Most of the output of the *diveRsity* function are graphs, however, also a table with the actual diversity values is given. The measure used to quantify diversity is the average heterozygosity, which is calculated as follows:

$$H_e = \frac{n_{He}}{n_n}$$

With H_e being the level of heterozygosity, n_{He} the number of heterozygous loci in the sample of interest and n_n, the total number of loci used. n_n is the same for every sample of the multisample VCF file.

```
data("breeds", package = "variantscanR")
breeds <- breeds
pkg <- "variantscanR"
vcf_file <- system.file("extdata", "SNPs.recode.subset.rename.vcf.gz", package = pkg)
sample_name <- "sample_9"
diversity <- diveRsity(vcf_file, breeds, sample_name)
```

```
## Scanning file to determine attributes.
## File attributes:
##   meta lines: 3311
##   header_line: 3312
##   variant count: 31
##   column count: 36
##
Meta line 1000 read in.
Meta line 2000 read in.
Meta line 3000 read in.
Meta line 3311 read in.
## All meta lines processed.
## gt matrix initialized.
## Character matrix gt created.
##   Character matrix gt rows: 31
##   Character matrix gt cols: 36
##   skip: 0
##   nrows: 31
##   row_num: 0
##
Processed variant: 31
## All variants processed
```

Reporting

As with the *variantfiltR* function, the not the entire VCF dataset was used in the worked-out example for the *diveRsity* function. However, the graphs of the real example are displayed below.

The first graph gives a basic overview of all the samples included in the multi-sample VCF file. The level of heterozygosity is shown on the y-axis and the x-axis displays the different breed populations.

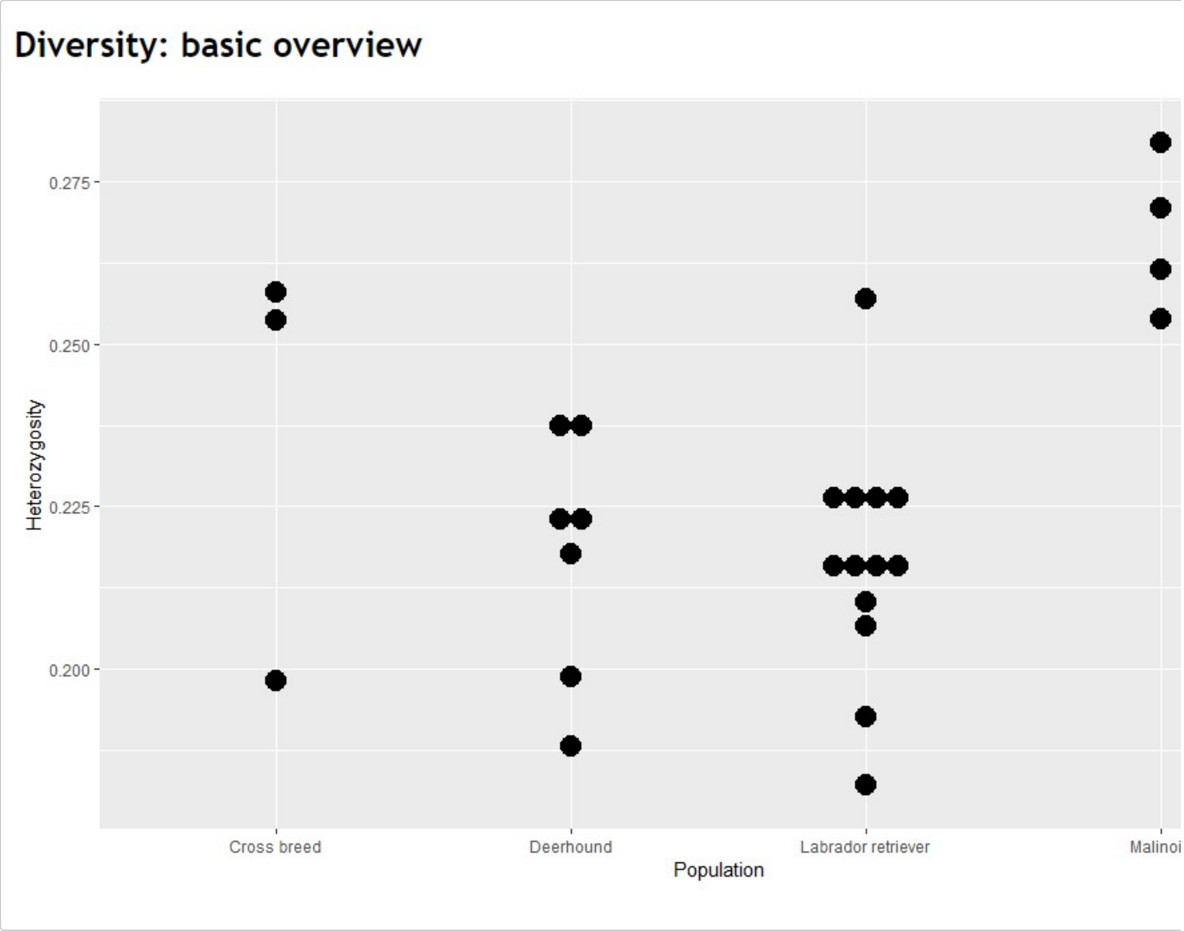

Figure 8: Basic overview

The second graph reveals the name of every sample.

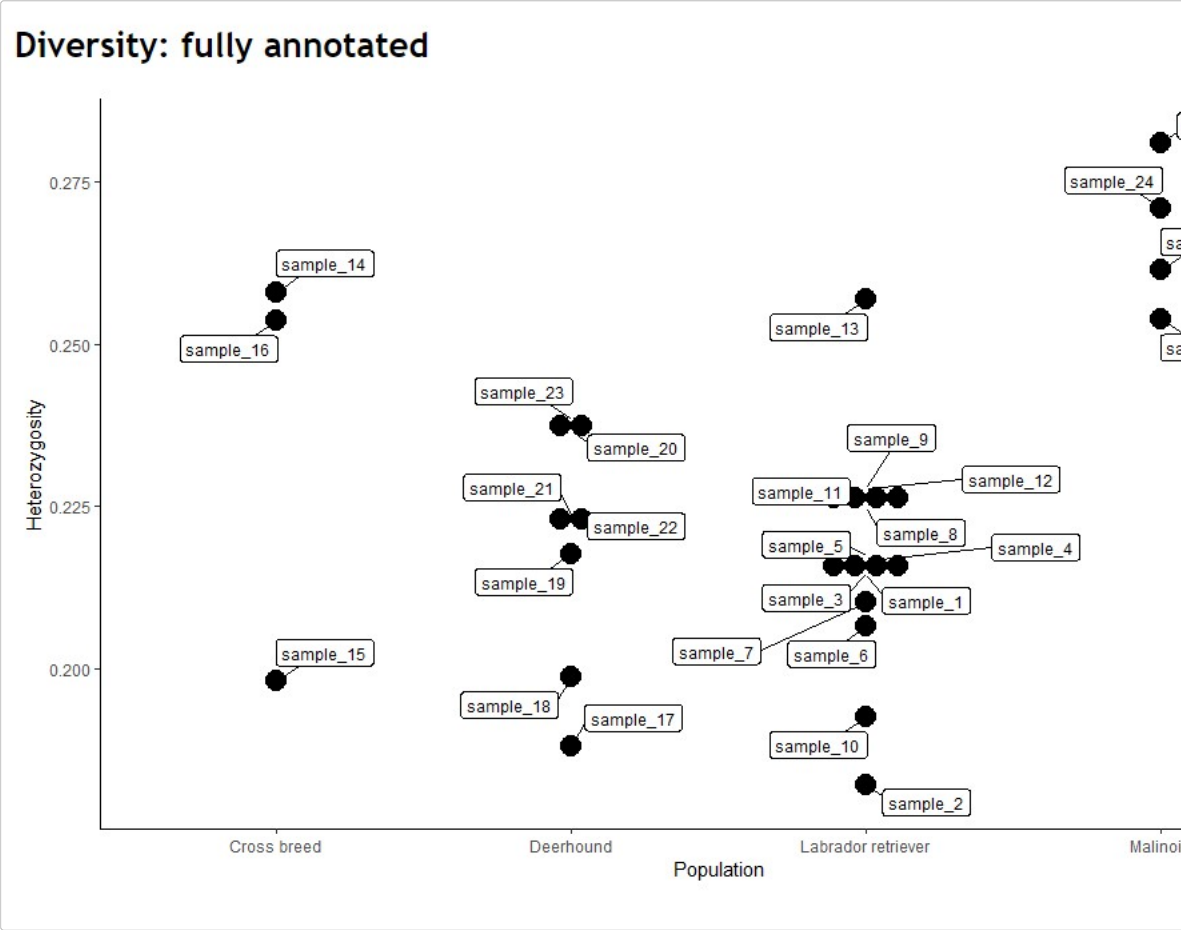

Figure 9: fully annotated

The third graph only reveals the name of the sample of interest, that was provided as a parameter to the *diveRsity* function.

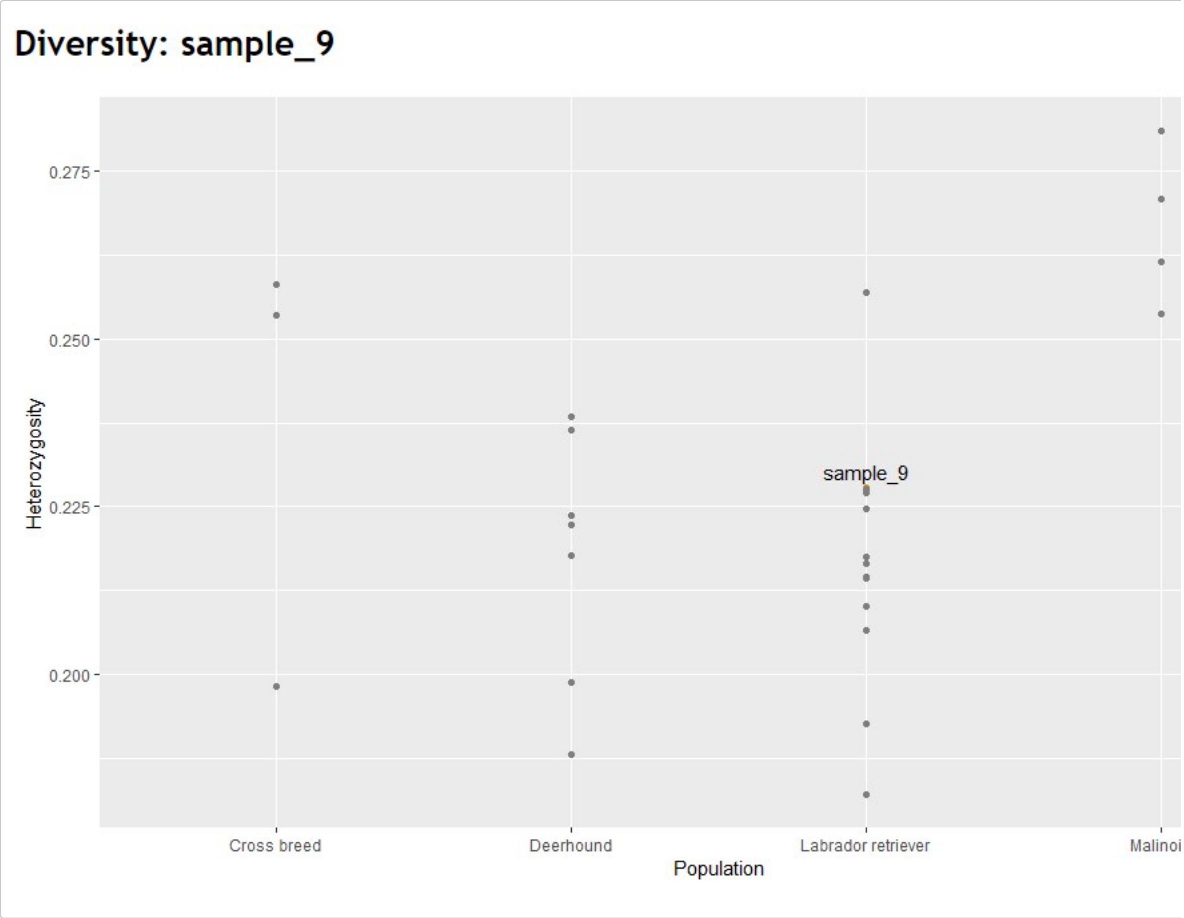

Figure 10

Lastly, the fourth graph only shows the highest sample of every breed.

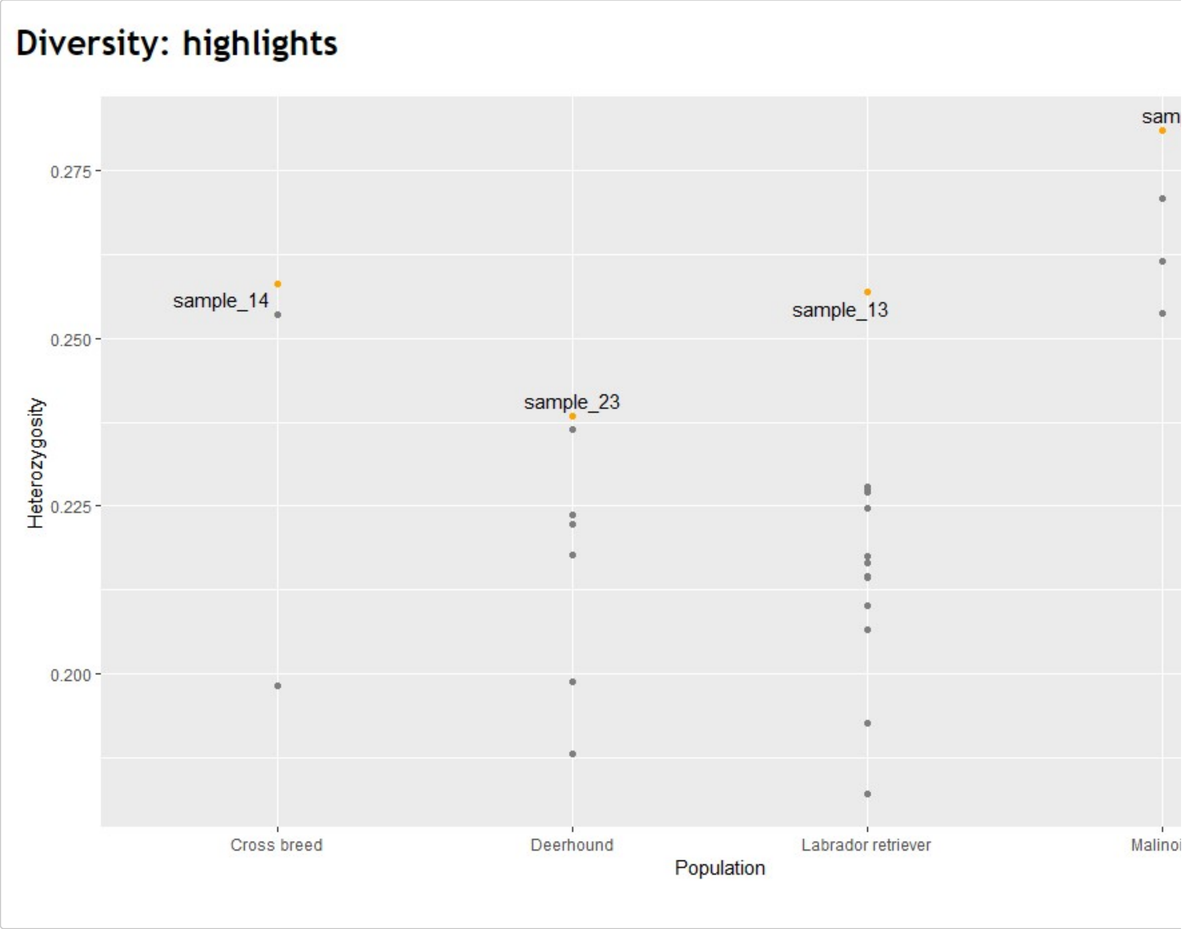

Figure 11

Next to the graphs, a table is included in the output of the *diveRstity* function, containing the heterozygosity values per sample.

Diversity

ID	Heterozygosity	Population
sample_1	0.214281872046519	Labrador retriever
sample_10	0.192590510668169	Labrador retriever
sample_11	0.227002181239191	Labrador retriever
sample_12	0.227755260121515	Labrador retriever
sample_13	0.256939756378982	Labrador retriever
sample_14	0.258045168595535	Cross breed
sample_15	0.198129674804401	Cross breed
sample_16	0.253600658406109	Cross breed
sample_17	0.188082795643977	Deerhound
sample_18	0.198734827477697	Deerhound
sample_19	0.217747379688925	Deerhound
sample_2	0.182008407587808	Labrador retriever
sample_20	0.236477527319281	Deerhound
sample_21	0.223598533647833	Deerhound
sample_22	0.22233040260135	Deerhound
sample_23	0.238404602387798	Deerhound
sample_24	0.270945678806478	Malinois
sample_25	0.28101138493896	Malinois
sample_26	0.261450160970611	Malinois
sample_27	0.253794307261563	Malinois
sample_3	0.214573690113419	Labrador retriever
sample_4	0.216573383502731	Labrador retriever
sample_5	0.217542972563723	Labrador retriever
sample_6	0.206621983986315	Labrador retriever
sample_7	0.210196419109915	Labrador retriever
sample_8	0.224656878434241	Labrador retriever
sample_9	0.227911255032853	Labrador retriever

Figure 12

extrafiltR

When there is genetic heterogeneity, the variant responsible for a certain phenotype might not be known and as such are not present in the VOI file. The disease-associated variant might however occur in genes known to be associated with the phenotype. The optional extrafiltR function collects all other variants present in the genes that contain the variants of interest as this might be valuable input in these cases. A dataframe is rendered in the R environment. This dataframe can be searched by the user to identify potentially interesting variants.

```
require(stringr)
require(svMisc)
vcf <- vcf_chr
variants_file <- variant_file_processed_1
BED_file_annot <- BED_file_fully_annotated
extra <- extrafiltR(vcf, variants_file, BED_file_annot)
```

```
## Progress: 161 on 161
```

```
head(extra)
```

```
##   Chromosome gene Location Wild_type
## 1       chr5 PKP1 63684323         A
## 2       chr5 PKP1 63684670         A
## 3       chr5 PKP1 63685053         G
## 4       chr5 PKP1 63685230         G
## 5       chr5 PKP1 63685760         A
## 6       chr5 PKP1 63685770         T
##                                                First_allele
## 1                                                         A
## 2                                                         T
## 3 A call cannot be made for this sample at this given locus
## 4 A call cannot be made for this sample at this given locus
## 5 A call cannot be made for this sample at this given locus
## 6 A call cannot be made for this sample at this given locus
##                                               Second_allele
## 1                                                         C
## 2                                                         T
## 3 A call cannot be made for this sample at this given locus
## 4 A call cannot be made for this sample at this given locus
## 5 A call cannot be made for this sample at this given locus
## 6 A call cannot be made for this sample at this given locus
##                         Zygosity
## 1                   Heterozygous
## 2                     Homozygous
## 3 Zygosity could not be determined
## 4 Zygosity could not be determined
```

```
## 5 Zygosity could not be determined
## 6 Zygosity could not be determined
```
