# Supplementary material for: VariantscanR: an R-package as a clinical tool for variant filtering of known phenotype-associated variants in domestic animals
Source: BMC Bioinformatics. 2023 Aug 1;24:305. doi: 10.1186/s12859-023-05426-6 (PMC10394849; doi:10.1186/s12859-023-05426-6)
Supplement: Supplementary file 2 — Additional file 2: Variants called in other samples. Description of data: Variants called in other samples of the multi-VCF file that are present in a wildtype homozygous state in the sample of interest. [file 12859_2023_5426_MOESM2_ESM.docx]

| Additional file 2: Variants called in other samples | | | | | | | | |
| --- | --- | --- | --- | --- | --- | --- | --- | --- |
| **Chromosome** | **Location** | **Gene** | **Reference** | **Allele 1** | **Allele 2** | **Zygosity** | **Inheritance** | **phenotype** |
| 11 | 33317810 | TYRP1 | T | T | T | Homozygous | Autosomal  recessive | Brown |
| 11 | 33326685 | TYRP1 | T | T | T | Homozygous |  | Brown |
| 13 | 61287796 | ADAMTS3 | C | C | C | Homozygous | Autosomal  recessive | Upper airway syndrome |
| 2 | 21731842 | SUV39H2 | A | A | A | Homozygous | Autosomal recessive | Nasal parakeratosis |
| 20 | 55850145 | MFSD12 | C | C | C | Homozygous | Autosomal recessive | White or cream |
| 24 | 23393552 | ASIP | C | C | C | Homozygous | Autosomal recessive | Recessive black |
| 30 | 37821686 | CYP1A2 | C | C | C | Homozygous | Autosomal recessive | Metabolizer of cognitive enhancer |
| 9 | 4188663 | PRCD | C | C | C | Homozygous | Autosomal recessive | Progressive rod-cone degeneration |
| 9 | 55282762 | DNM1 | C | C | C | Homozygous | Autosomal recessive | Exercise-induced collapse |
| M | 14474 | CYTB | G | G | G | Homozygous | Mitochondrial | Leucodystrophy |
| Additional file 3: Variants called in other samples of the multi-VCF file that are present in a wildtype homozygous state in the sample of interest | | | | | | | | |
