# Supplementary material for: VariantscanR: an R-package as a clinical tool for variant filtering of known phenotype-associated variants in domestic animals
Source: BMC Bioinformatics. 2023 Aug 1;24:305. doi: 10.1186/s12859-023-05426-6 (PMC10394849; doi:10.1186/s12859-023-05426-6)
Supplement: Supplementary file 4 — Additional file 4: File attributes provided by vcfscanneR function. Original lay out was preserved. Description of data: VCF file attributes provided by the vcfscanneR function after uploading of the VCF file into the R environment. Original lay-out is preserved. [file 12859_2023_5426_MOESM4_ESM.docx]

| *Additional file 4: File attributes of VCF file provided by vcfscanneR function.* | |
| --- | --- |
| **File attributes:** |  |
| - meta lines | 3311 |
| - header_line | 3312 |
| - variant count | 4001471 |
| - column count | 13 |
| **Meta line 3311 read in.** |  |
| **All meta lines processed.** |  |
| **gt matrix initialized** |  |
| **Character matrix gt created.** |  |
| - Character matrix gt rowns: | 4001471 |
| - Character matrix gt cols: | 13 |
| - Skip: | 0 |
| - nrows: | 4001471 |
| - row_num: | 0 |
| **Processed variant:** |  |
| All variants processed |  |
