# Supplementary material for: VariantscanR: an R-package as a clinical tool for variant filtering of known phenotype-associated variants in domestic animals
Source: BMC Bioinformatics. 2023 Aug 1;24:305. doi: 10.1186/s12859-023-05426-6 (PMC10394849; doi:10.1186/s12859-023-05426-6)
Supplement: Supplementary file 5 — Additional file 5: Comparison with other tools. Description of data: A comparison between all tools found in a Pubmed search for the following 4 search terms: VCF, variant filtering, standardised reporting and animal. [file 12859_2023_5426_MOESM5_ESM.docx]

| *Additional file 5: comparison between all tools found in a PubMed search for the following 4 search terms: VCF, variant filtering, standardized reporting and animal.* | | | | | | |
| --- | --- | --- | --- | --- | --- | --- |
|  | Handles VCF file | Allows Non-human samples | Clinical settings goal | Standardised reporting | Identifying known  Variants | Diversity measures |
| webGQT | x | x | x |  |  |  |
| VariFAST | x |  |  |  |  |  |
| vcfView | x |  |  |  |  |  |
| VCF-Server | x |  |  |  |  |  |
| VCF/Plotein | x |  |  |  |  |  |
| VCF-Miner | x | x |  |  |  |  |
| VCF.Filter | x |  | x |  |  |  |
| FIREVAT | x |  |  |  |  |  |
| DaMold | x |  | x |  |  |  |
| BrowseVCF | x |  | x | x |  |  |
| **VariantscanR** | **x** | **x** | **x** | **x** | **x** | **x** |
